# Swin-YOLO-SAM: a hybrid Transformer-based framework integrating Swin Transformer, YOLOv12, and SAM-2.1 for automated identification and segmentation of date palm leaf diseases

**DOI:** 10.3389/fpls.2025.1666374

**Published:** 2025-09-15

**Authors:** Ali Saeed Alzahrani, Abid Iqbal, Wisal Zafar, Ghassan Husnain

**Affiliations:** ^1^ Department of Computer Engineering, College of Computer Sciences and Information Technology, King Faisal University, Al Ahsa, Saudi Arabia; ^2^ Department of Computer Science, Cecos University of Information Technology (IT) and Emerging Sciences, Peshawar, Pakistan

**Keywords:** date palm disease, plant disease detection, severity prediction, zero-shot segmentation, automated crop monitoring, Grounding DINO, Segment Anything Model (SAM)

## Abstract

The cultivation of date palm (*Phoenix dactylifera* L.) is acutely impacted by numerous fungal, bacterial, and pest-related diseases that diminish yield, spoil fruit quality, and undermine long-term agricultural sustainability. The traditional methods of monitoring diseases, which rely heavily on expert knowledge, are not scalable and depend heavily on classical models that do not generalize readily to real-world conditions. Recent improvements in deep learning over the last two decades, particularly with Convolutional Neural Networks (CNNs), have led to significantly greater automation. However, CNNs still require relatively large labeled datasets and struggle with ambiguous or complex background features, small lesions, and overlapping symptoms when diagnosing plant diseases. To address these difficulties, we introduce an innovative hybrid Transformer deep learning framework based on four sophisticated modules: 1) Swin Transformer for hierarchical image classification, 2) YOLOv12 for real-time detection, 3) Grounding DINO with SAM2.1 for zero-shot segmentation, and 4) Vision Transformer (ViT) and a regression head for predicting disease severity. This collective architecture can deliver accurate detection, the most accurate segmentation, and quantification of disease severity in real-world, low-annotation-based scenarios and adverse environmental context situations. Experimental findings on a curated dataset of 13,459 palm leaf images show that the proposed model outperforms all previous CNN-based models with a classification accuracy of 98.91%, a precision of 98.85%, a recall of 96.8%, and an F1-score of 96.4%. These results represent a new standard for automated, scalable, and interpretable disease diagnosis in precision agriculture.

## 1 Introduction

The date palm (*Phoenix dactylifera* L.) is of significant economic, social, and cultural importance in arid and semi-arid regions of the world, as it is a staple food and a major part of agricultural economies. The date palm provides livelihoods for farmers and laborers and food security for communities and is an important part of the cultural heritage for many people. Crop productivity and longevity of date palm orchards are constantly faced with a wide array of diseases that infect the leaves, generally fungi, bacteria, and phytoplasmas, which are responsible for yield loss, reduced fruit quality, or total tree mortality. Therefore, diseases that affect date palms present a serious hindrance to sustainable date palm production. The unfortunate history of date palm cultivation has been marked by devastating disease outbreaks. One of the diseases that has had the most historical impact is Bayoud (Fusarium wilt), which is caused by the soil-borne fungus *Fusarium oxysporum* f. sp. *albedinis*. Bayoud was first reported in Morocco in 1870 and has, over the past century, wiped out millions of date palms in North Africa, especially in Morocco and Algeria; caused the destruction of two-thirds of Morocco’s best date palm producers; and has sped up desertification (Ahmed and Ahmed, 2023). Bayoud attacks both mature and immature palms and offshoots. It begins as a whitening and drying of the fronds but progresses quickly and results in the death of the entire palm. The serious impact of Bayoud certainly emphasizes a long-standing and urgent need for disease management strategies. Two additional diseases of concern are Khamedj (inflorescence rot caused by *Mauginiella scaettae*), which has impacts on productivity, and black scorch (*Thielaviopsis paradoxa*), which results in necrosis in various parts of the tree (Kamal et al., 2025). There always exists the threat of disease phases in date palm cultivation.

The traditional approach to identifying and monitoring diseases of date palm leaves is largely dependent upon manual visual inspection by agricultural experts and farmers, which is fundamental and relies on valuable expert knowledge, but is also labor-intensive, slow, and inherently prone to human error or subjectivity. The scale of date palm plantations, the often deceptive and developmentally occurring nature of early disease symptoms, and the necessity of ongoing, systematic monitoring complicate such difficulties. The cultural control practices include orchard sanitation, such as removing and burning diseased plant tissues (leaves, inflorescences, and offshoots) to limit pathogen movement. Irrigation and drainage are also important, as other fungal pathogens may thrive in excessive humidity (Alhudaib et al., 2022). The chemical control application of fungicides or bactericides to infected palms or the surrounding soil has traditionally been used to minimize disease spread. Nevertheless, chemical mitigations are typically harmful to the environment and are dependent on the concentration of the compound. Also, deep-seated pathogens or resistant lineages may resist chemical controls entirely (Namoun et al., 2024). Researchers and clonal programs have often bred and cultivated disease-resistant cultivars of date palms for long-term problems like Bayoud; however, obtaining high-quality resistant cultivars can take decades. Although these orderly approaches provide limited levels of control, their scalability, efficiency, and real-time responsiveness are severely undermined, especially regarding potential widespread or newly emerging biological assaults where pests and pathogens are a matter of concern and the increasing global demand for date palm products (El Bouhssini and Faleiro, 2018).

Recent advances in artificial intelligence (AI) and its subdivisions, such as machine learning (ML) and deep learning (DL), have provided new resources in multiple fields, including agricultural diagnostics. These computer algorithms offer higher levels of automation and enhanced levels of accuracy in the detection and classification of diseases in plants. The initial applications of ML in plant disease detection relied upon handcrafted features obtained from leaf image data (e.g., color, shape, and texture) to classify healthy and diseased samples using some basic classifiers [e.g., Support Vector Machines (SVMs), K-Nearest Neighbors (KNN), and Decision Trees], as conducted by Slika et al., (2023). Initial methods showed promise; however, they were significantly hindered by the need for domain knowledge to accurately carry out feature engineering and the sheer variability and complexity intrinsic to real-world agricultural images (urban environments, varying lighting, background clutter, disease signs, etc.). The emergence of deep learning, specifically Convolutional Neural Networks (CNNs), represents a defining step change in the field of automated plant disease diagnosis. More specifically, CNNs can extract and learn classification features directly from raw image data, meaning that the manual feature extraction step is no longer a consideration for the researcher. This ability to learn features equates to the identification of features that represent a more abstract representation of disease symptoms. Several studies have successfully applied CNNs to palm disease classification, with high accuracy rates in classifying between healthy and various diseased leaf samples (Slika et al., 2023; Alu’datt et al., 2025). CNNs have rapidly become the foundation of modern plant disease detection systems as a result of their unparalleled utility for image-based detection. In addition to standard CNNs, AI in plant pathology continues to grow, as it incorporates neural networks with more complex architectures and technologies to improve diagnostic function. Neural network techniques such as CNNs comprise a group of neural networks, but the term neural network can include other architectures and more complex configurations. The incorporation of hybrid architectures, such as combining CNNs with Recurrent Neural Networks (RNNs) for time series data, can offer a more thorough understanding of disease progression over time. There are continuing advancements in neural network research, which will provide ample opportunities to improve the efficiency and accuracy of plant disease detection (Namoun et al., 2024). Transformer techniques, such as Vision Transformers (ViTs), were originally proposed for natural language processing, and they have been found to outperform many traditional computer vision methods in numerous applications. Vision Transformers use self-attention techniques to learn complex patterns of images, such as long-range dependencies across the entire image. This is a valuable asset when working with disease symptoms that can be subtle and distributed across large date palm leaves (Liu and Zhang, 2025). They are advantageous because they can model relationships globally across the image, which can lead to a more robust model for disease classification and detection, in many cases surpassing CNNs, which may be limited by relevance maps among complex conditions. Leveraging those contemporary advancements, this research introduced a fully developed state-of-the-art method for the automatic diagnosis and management of infected date palm leaves. Our unified approach integrates several very successful and complementary techniques to solve different aspects of the disease management: to classify diseases accurately, we employed the Swin Transformer, which builds on traditional ViTs but constructs hierarchical feature maps and makes use of shifted window attention, which enhances computational efficiency and scalability for high-resolution images while combining the global context understanding of transformers (Karthik et al., 2024). This architecture is a particularly good fit for two-dimensional images like date palm leaves, where it can capture fine-grained local symptoms as well as larger patterns associated with the disease to identify the disease as accurately as possible. For the detection and localization of affected areas in date palm images in real-time, you only need to look once (YOLO). YOLO series models are known for their speed and accuracy in object detection, which suits practical, on-field applications where quickly identifying the diseased spot or entire infected leaves will enable early and timely intervention (Alif and Hussain, 2025). YOLOv12’s employed detection capabilities will improve the prospects of successfully scanning date palm orchards. To define disease regions (segmentation) in a precise (accurate) manner, without first using a certain amount of pixel-level annotation for each disease, we recommend using zero-shot segmentation in combination with Segment Anything Model 2.1 (SAM2.1). Grounding DINO is a powerful, zero-shot object detection model that can detect an object based on textual prompts, while SAM2.1 is a very powerful segmentation model that can segment any object with the right cues. When these two models are combined, textual descriptions of disease symptoms (“brown spots” and “chlorotic areas”) can be used to generate accurate segmentations of these regions (even for unseen disease types) and to reduce the annotation workload immensely. It has the potential to provide flexibility and adaptability to new levels and types of diseased areas for identification and quantification. Severity prediction using ViT + Regression Head is one thing to classify the disease, but another to understand how serious that disease is, so we can make informed treatment decisions and prognosticate. For this component, we will build a ViT with a regression head to predict disease severity. The ViT backbone will extract informative visual features from infected leaf images, and the regression head will not classify the images into discrete classes but instead produce a severity score on a continuous scale. This way, we can obtain more granular information about the severity of the disease to make more informed treatment decisions, track efficacy over time, and still record the disease progression as it develops.

The goal of this study was to develop a reliable, explainable, and effective deep learning framework for the early detection and management of palm diseases. Here, the main innovation is the combination of four cutting-edge approaches under a single pipeline: 1) classification of disease with Swin Transformer, 2) zero-shot detection of infected areas with Grounding DINO, 3) lesion segmentation with SAM2.1, and 4) severity prediction using Vision Transformer with a regression head. With this framework, we aim to achieve more accurate detections in earlier stages and complex environments than traditional CNN models, leveraging fine-grained appearance-based features. Our work introduces several key innovations:

- A robust AI framework that combines classification, detection, segmentation, and severity assessment, specifically designed for palm disease identification.
- Engineered zero-shot learning and automated segmentation that remove the need for annotated datasets, making the solution feasible in low-resource constraints.
- Performance quality is accentuated across both the controlled images taken in laboratories and images captured in applied field conditions. We demonstrate high scalability and adaptability for precision agriculture.
- Able to detect subtle symptoms in difficult conditions (e.g., tiny lesions, overlapping fronds, and dust), which points to the current system’s limitations.

## 2 Related works

Palm disease detection has transitioned from human visual inspection processes to deep learning technologies. This section will provide a comparative review of the palm disease detection methods, including manual, traditional ML, CNN-based models, transformer-based models, and segmentation methods. For each discussion, we will explain the advantages and disadvantages of each method and the need for a single hybrid palm disease detection solution. **Table 1** presents a summary of the literature review.

**Table 1:** Literature review summary for date palm classification, detection, and segmentation systems.

Author and reference	Methodology	Performance	Key limitation	Contribution to justifying the hybrid model
(Santhosh Kumar and Jayanthi, 2024)	Manual visual inspection	N/A	Labor-intensive, subjective, ineffective for early detection	Highlights the need for automation and early detection
(Hadjraoui et al., 2017)	Traditional ML (SVM and KNN)	Low robustness	Dependent on handcrafted features; poor generalizability	Shows ML limitations in field variability
(Irshad et al., 2023)	CNNs (ResNet and Inception ResNet)	99.62% accuracy	Not scalable in low-resource settings; requires labels	Demonstrates CNN strength but lacks scalability
(Rybacki et al., 2024)	CNNs (MobileNetV2 and DenseNet)	96.99% accuracy, F1: 0.97	N/A	Strong classification, but not suitable for segmentation
(Shehu et al., 2025)	YOLOv5/YOLOv12	mAP 80%–90%	Real-time, but lacks segmentation precision	Supports object-level detection capability
(Liu and Zhang, 2025)	Swin Transformer	Up to 97.6% accuracy	Limited use in palm diseases	Justifies the Swin Transformer for complex feature detection
(Hessane et al., 2025)	Zero-shot segmentation (DINO + SAM)	OA: 96.56%, mIoU: 81.5%	Solves annotation issue	Enables scalable, annotation-free segmentation
(Hua et al., 2025)	Hybrid CNN (ECA-Net + ResNet + DenseNet)	~99% accuracy	N/A	Supports a hybrid approach for improved accuracy
(Imran et al., 2025)	CNNs for Fusarium wilt	Precision: 91%, recall: 94%	Class imbalance, visual similarity	Confirms CNN effectiveness but is limited in nuance
(Sun et al., 2024)	UAV + DL + remote sensing	Accuracy: 85%–95%	High cost, infrastructure-heavy	Remote sensing is helpful, but not scalable
(Moses, 2024)	CNNs	94% (recall) Overall F1-score: 90%	Class imbalance, variations in images, visual similarities of diseases	-
(Kamal et al., 2025)	AI prediction system (PDS)	R^2^: 77%–90%	N/A	Adds interpretability insights for pest monitoring

ML, machine learning; SVM, Support Vector Machine; KNN, K-Nearest Neighbors; CNNs, Convolutional Neural Networks.

Kumar conducted a manual inspection as the traditional approach, which is extremely tedious, labor-intensive, and inherently subjective, especially for large plantations. It cannot detect early symptoms, which are usually relatively subtle and are instead missed (Santhosh Kumar and Jayanthi, 2024).

Conventional ML methods are an improvement over manual methods, as ML methods use handcrafted features (like color, texture, and shape) and classifiers (like SVM, KNN, and Decision Trees). Although they were better than manual methods, they were only as good as the features that an operator crafted and did not generalize well enough under varying field conditions (Hadjraoui et al., 2017).

DL, especially convolutional neural networks (ConvNets) or CNNs, delivered a great improvement in accuracy and automation. One study using ResNet and Inception ResNet achieved classification accuracies greater than 97.8%, a higher accuracy than that of ML baselines for palm diseases. The same pattern occurred using MobileNetV2, DenseNet, and InceptionV4, all achieving F1-scores above 97%, showing significant reliability for designating the type of disease (Irshad et al., 2023).

Rybacki et al. further used the power of CNNs in date palm disease classification. They implemented pre-trained architectures InceptionV4, DenseNet, and MobileNetV2. Their results indicated that MobileNetV2 had an overall classification accuracy of 96.99% and a macro-average F1-score of 0.97 across nine separate categories of date palm leaf conditions, implying an overall high degree of accuracy and reliability. Similar to other research in the field, these results consistently demonstrate that CNN-based models outperform conventional ML methods, achieving accuracies above 95% (Rybacki et al., 2024).

Plant disease detection and localization tasks utilizing object detection models (e.g., YOLOv5 and YOLOv12) have demonstrated mean Average Precision (mAP) scores between 80% and 90% in agricultural settings. Object detection models such as YOLO represent opportunities for applying real-time detection methods, but they still require manual bounding box annotations and lack semantic segmentation accuracy (Shehu et al., 2025).

In addition to classical CNNs, state-of-the-art deep learning architectures, primarily Transformers, are becoming a strong alternative. While Swin Transformer-specific applications for date palm leaf disease classification are emerging, many sources concerning other plant diseases show that they perform quite robustly. One application of an Efficient Swin Transformer for general plant disease classification produced a precision of 80.14% and a recall of 76.27% on the PlantDoc dataset, which was better than regular Swin-T (Liu and Zhang, 2025). Furthermore, a dual-track feature fusion model containing the Swin Transformer for grape leaf diseases produced an accuracy of 97.6%, with precision, recall, and F1-score also containing an accuracy of 96.60% (Makhija and Scholar, 2025). This reinforces that the Swin Transformer can capture complex features to provide intricate precision based on plant disease classification.

The area of Explainable AI (XAI) is developing to increase trust and transparency in deep learning models. Different authors have provided different XAI techniques, including Grad-CAM and LIME, tracking the model’s diagnostic decisions back to the regions of an image that contributed to the model’s decision, thus leading farmers to explain, trust, and understand the outputs of information from an AI (Hua et al., 2025).

One of the main challenges to implementing DL, especially for segmentation tasks, is the large demand for pixel-level annotations. Several studies have shown that zero-shot segmentation, using open-set detectors (like Grounding DINO) and large, promotable segmentation models (like SAM), can be a game-changer (Bao et al., 2025). Together, these models enable accurate mapping of the boundary of objects (e.g., areas of disease) based on textual descriptions given as multi-prompting and entirely bypassing the need to painstakingly annotate every instance of a disease, pixel by pixel (Namoun et al., 2024). For example, R et al. (2025) used Grounding DINO and SAM for unsupervised fine segmentation of desert vegetation and reported an overall accuracy of 96.56% and a mean Intersection over Union (mIoU) of 81.50% (R et al., 2025).

The study presented a hybrid model that combines the Efficient Channel Attention Network (ECA-Net) and ResNet50 and DenseNet201 to identify palm leaf diseases, achieving training and validation accuracies of 99.54% and 98.67%, respectively. This indicates that researchers are still developing hybrid CNN architectures, which can yield improved performance (Hessane et al., 2025).

Daranagama et al., (2025), Dubas-Infested Date Palm Trees, exemplifies the application of advanced remote sensing techniques, utilizing very high resolution (VHR) satellite imagery and Unmanned Aerial Vehicle (UAVs), integrated with highly sophisticated deep learning. Their system achieved an overall accuracy of 87% for detecting palm trees and an accuracy of 85% when assessing health levels, also showing high- and low-level accuracies of over 95% and 93%, respectively, for infestations (Daranagama et al., 2025).

In terms of Fusarium wilt detection, it was reported that CNNs were able to identify Fusarium wilt, Ganoderma rot, and additional fungal infections in palm trees. The study reported a precision of 91% and a recall of 94% in terms of Fusarium wilt detection, contributing to an overall F1-score of 90%. The study noted challenges such as class imbalance, variations in images, and visual similarities of diseases (Imran et al., 2025).

Zhao et al. explored an AI-powered prediction system for Parlatoria Date Scale (PDS), specifically for White Scale Disease (WSD), and applied machine learning techniques to images of leaflets, achieving R^2^ scores of between 77% and 90% for predicting population incidence. This study also highlights the importance of model interpretability, and LIME and SHAP for robust pest management (Zhao et al., 2025).

## 3 Hybrid Transformer framework for palm disease detection and analysis

**Figure 1:**
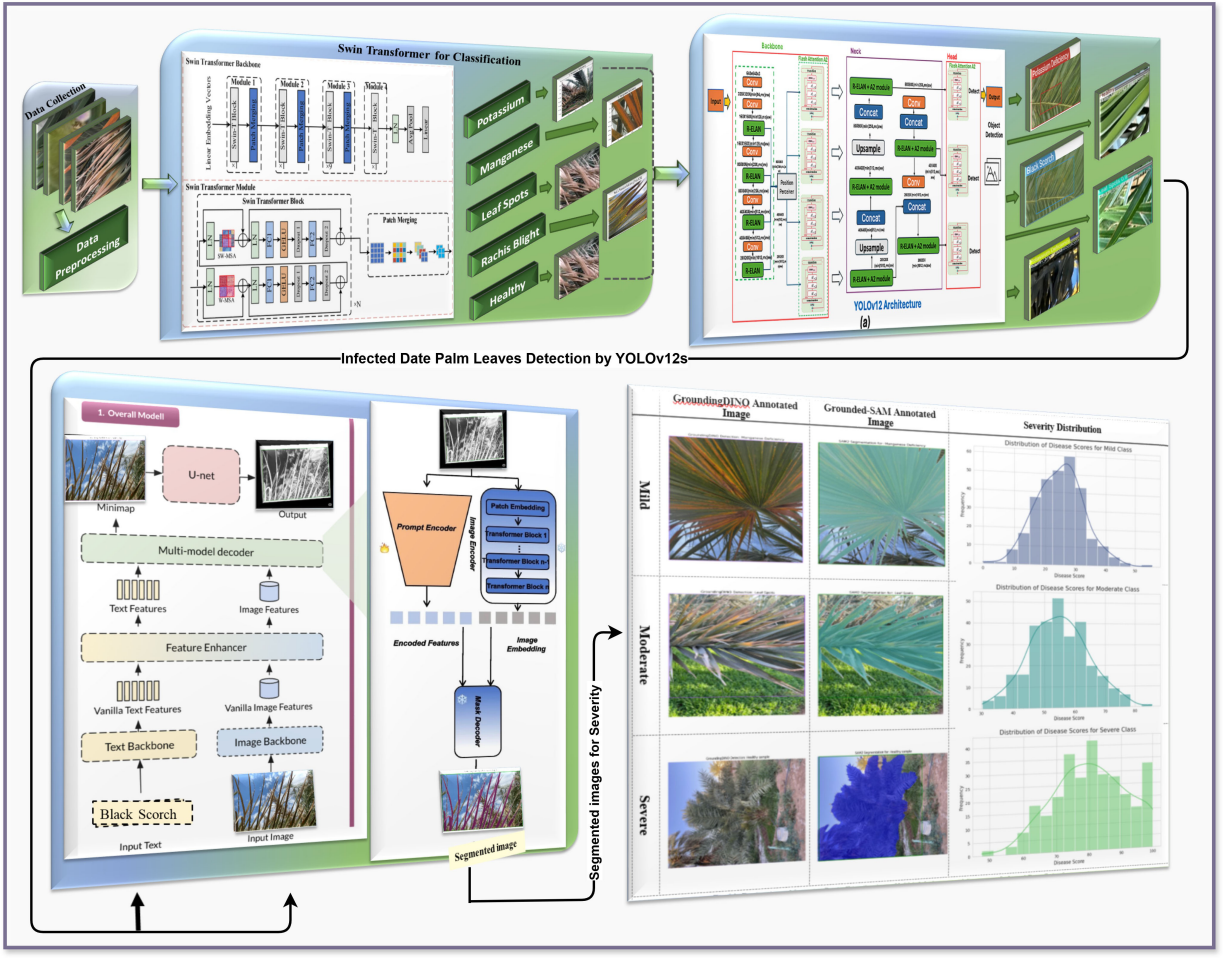
Hybrid Transformers-based proposed methodology for infected date palm leaf classification, detection, and segmentation.

In this study, we developed a comprehensive, multi-tiered, deep learning pipeline (**Figure 1**) for automating palm leaf disease classification, detection, segmentation, and severity prediction.

The pipeline begins with data acquisition and preprocessing, which involves obtaining palm leaf images and processing them through resizing, normalization, and augmentation to ensure consistent and adaptable inputs for learned generalization.

Next, we developed a Swin Transformer-based classification module using the preprocessed images. The Swin Transformer features hierarchical self-attention and shifted window techniques to extract rich spatial representations from the palm leaves effectively. We used the classification module to classify the palm leaves using five leaf disease categories: *Pestalotiopsis*, anthracnose, leaf spots, rachis blight, or healthy (Liu and Zhang, 2025).

After the initial classification, we employed a severity prediction module for palm leaf disease severity prediction, specifically, a ViT with a regression head. This module helps capture fine-grained features and report the results as continuous severity scores, which are mapped into three classes: mild, moderate, and severe. The severity prediction module is useful for quantitatively understanding the magnitude of the diseases and ideally implementing an early degree of planning, intervention, and treatment while still being scalable to explore other crop disease applications (Dosovitskiy et al., 2021).

The localization of the diseases was conducted using a YOLOv12 object detection framework whose single-stage architecture contained multiple-level detection layers, which utilize accurate bounding box prediction while having swift inference times suitable for reliable detection of affected areas in challenging field environments (Alif and Hussain, 2025).

Lesion-level accuracy is achieved through further refinement of detected regions with a segmentation module that follows the integration of Grounding DINO and Segment Anything Model v2 (SAM2). Grounding DINO leverages text-based prompts to facilitate zero-shot detection. SAM2 provides pixel-level segmentation of infected area pixel(s), which eliminates the need for pixel-level annotations and allows the detection of rare or early-stage infections (Skalski, 2023).

In summary, the proposed end-to-end framework brings classification, severity estimation, detection, and segmentation together into a single pipeline. An end-to-end technique has been demonstrated using Swin Transformer, ViT with a regression head, YOLOv12, Grounding DINO, and SAM2 to form a one-stop shop for concerns involved in palm leaf disease identification, analysis, and monitoring while encouraging precision agriculture and preemptive disease management.

## 4 Materials and methods

### 4.1 Data collection and preprocessing techniques

#### 4.1.1 Dataset collection

The current article proposes an image dataset of palm leaf diseases to facilitate the early detection and classification of date palm infections. The dataset consisted of images of eight primary types of disorders of date palm leaves: three physiological, four fungal, and one pest-related. Specifically, the sampled images showed symptoms and/or signs of potassium deficiency, manganese deficiency, magnesium deficiency, black scorch, leaf spots, Fusarium wilt, rachis blight, and *Parlatoria blanchardi*. The dataset also included a baseline of healthy palm leaves. A total of 608 raw images were collected over 3 months, covering the autumn and spring seasons, from 10 actual date farms in the Madinah Region of Saudi Arabia. The images were collected using a smartphone or SLR camera, focusing solely on purely inflected leaves and leaflets. Date palm fruits, trunks, and roots were not included in this dataset. The infected leaf images were filtered, then cropped and augmented, labeled/trained, and finally classified into their respective disease categories. Our processed dataset contained 3,089 images. Our dataset can be used to train classification deep learning models of infected date palm leaves (Namoun et al., 2024). The images were taken using six smartphones and one SLR camera, and all were recorded as raw images, with pixel resolutions ranging from 2778 × 1284 to 6000 × 4000. The pixel resolution of the processed images, however, was limited to 300 × 300 pixels. All leaf images from our dataset were taken from a distance of 15 to 100 cm. Images of infected palm leaves were taken at different angles and under different lighting conditions. These variations of palm leaf infection provide rich training and testing capabilities for smart palm disease detection and management systems. The contents of our dataset are made available in the Mendeley Data Repository (Namoun et al., 2024) with the following palm leaf conditions and pathologies. Image acquisition was conducted under controlled and field conditions. The palm leaf images in the controlled condition were taken under standardized conditions, including fixed camera distance, angle, and consistent lighting, to eliminate variability and noise during classification and training. The field-condition images were purposely collected with variability in camera angle, distance, and lighting (including shadows, dust, and overlapping fronds). The variability in these images was retained on purpose to simulate natural scenarios and improve the robustness and generalizability of the proposed framework for intended use in precision agriculture. The sample of each disease category is shown in **Table 2**.

**Table 2:** A sample of date palm leaf diseases/disorders and a healthy set.

Leaf disorder	Example 1	Example 2
K-Deficient	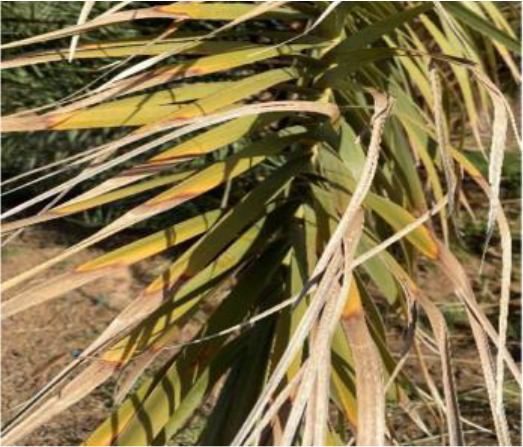	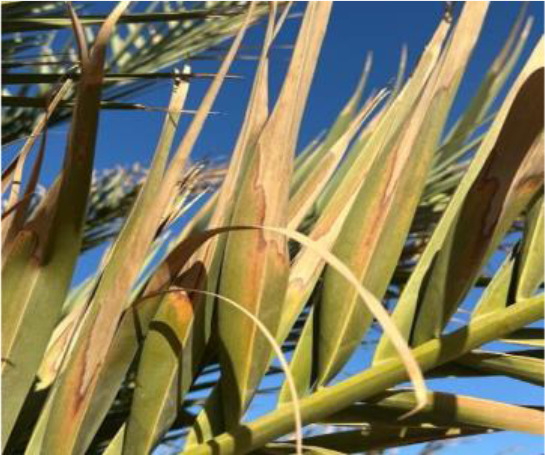
Mn-Deficient	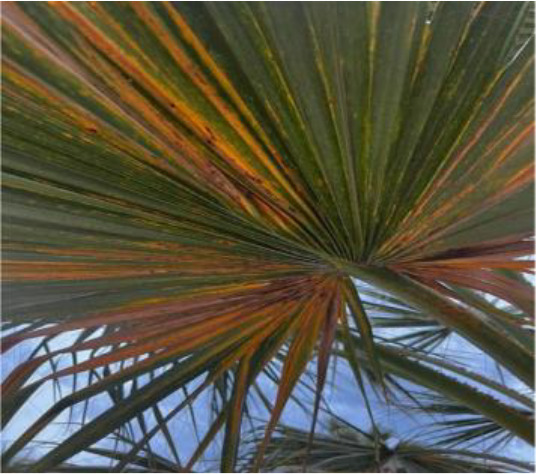	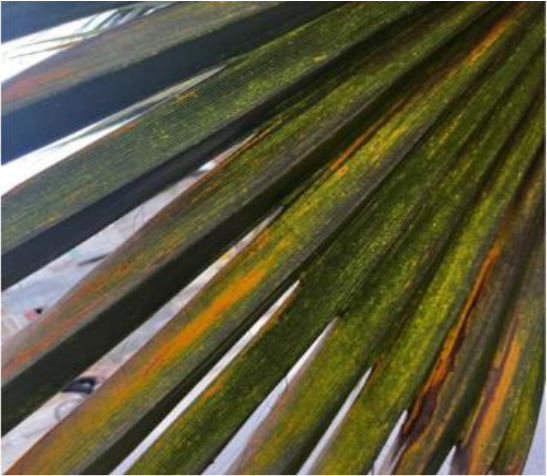
Mn-Deficient	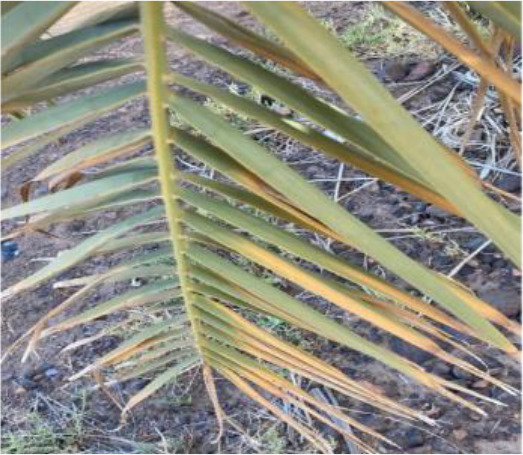	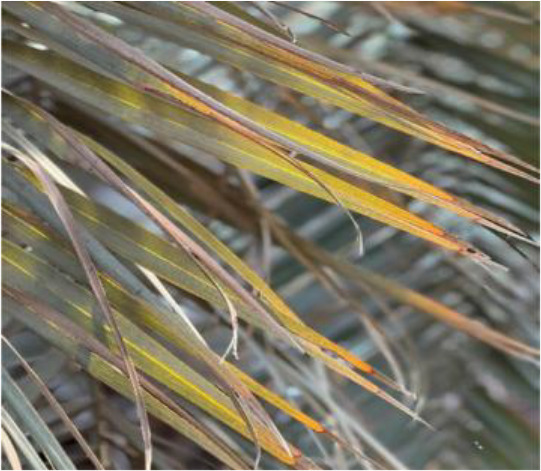
Black scorch	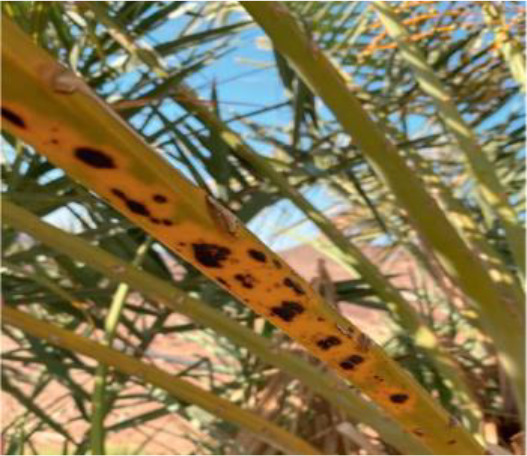	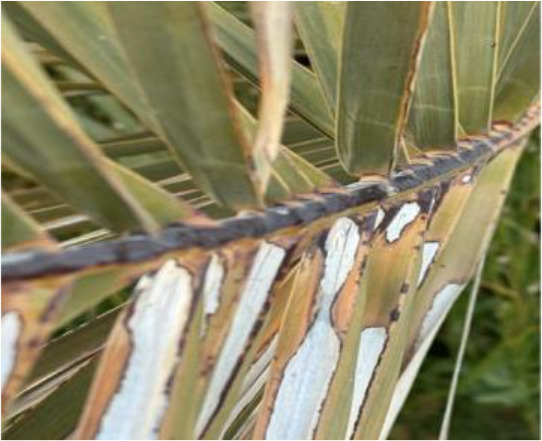
Leaf spots	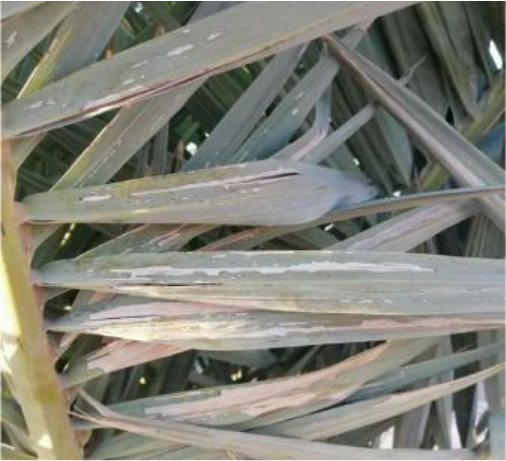	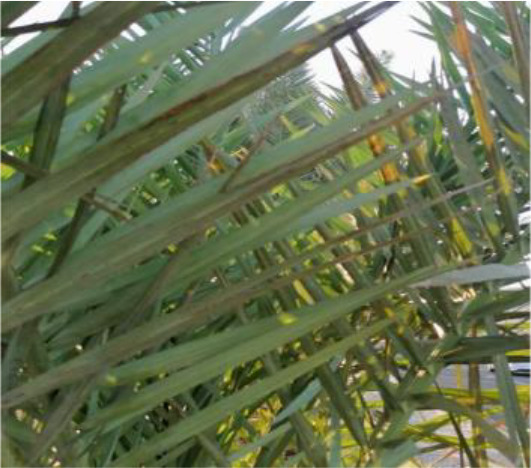
Fusarium wilt	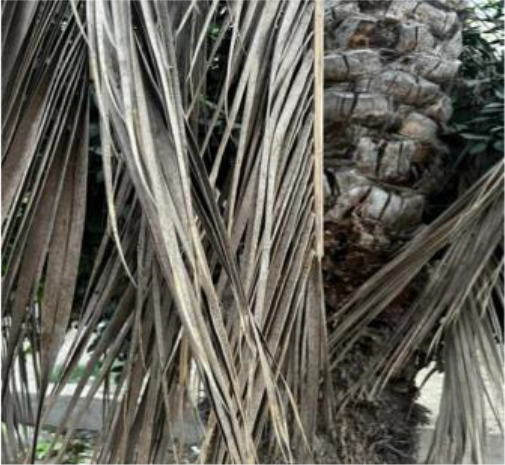	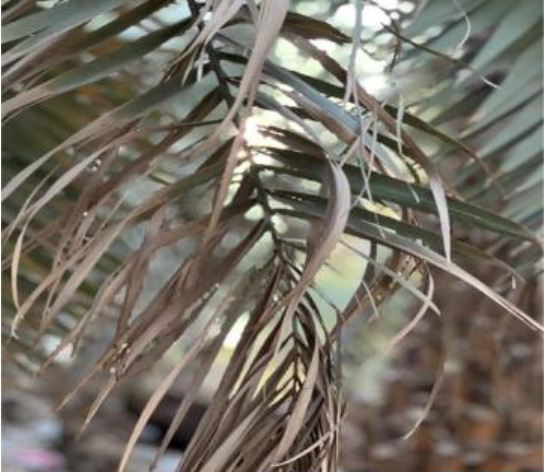
Rachis blight	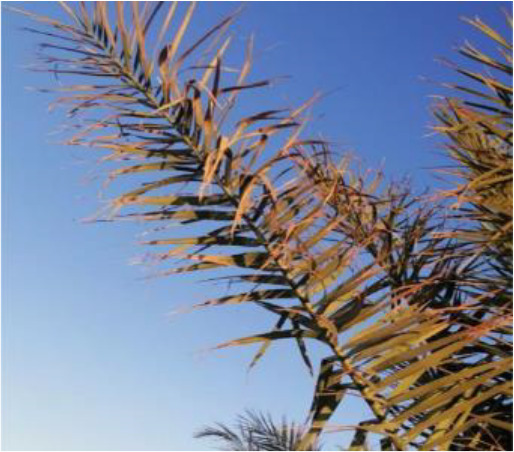	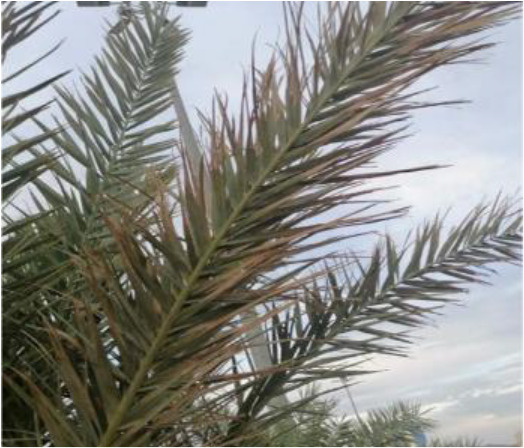
Parlatoria blanchardi	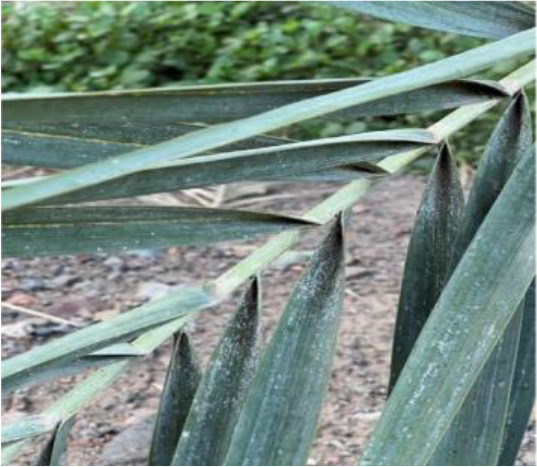	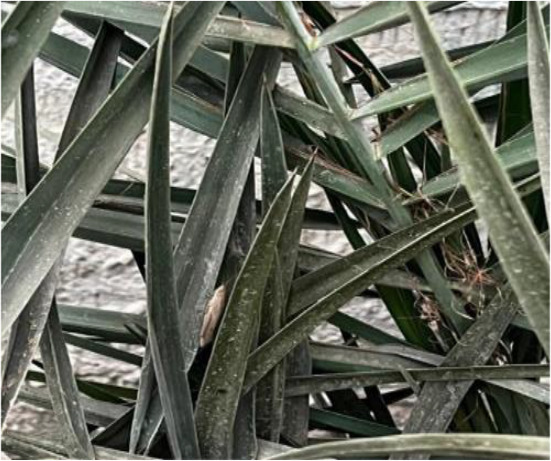
Healthy	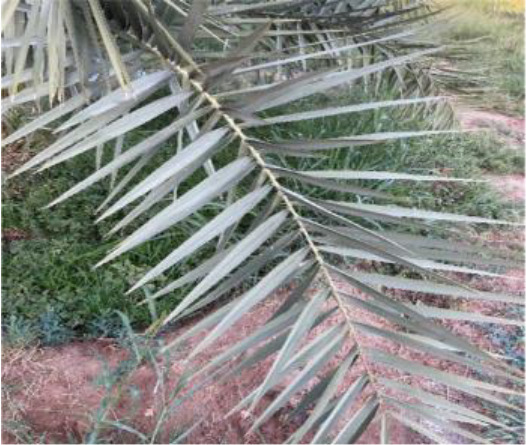	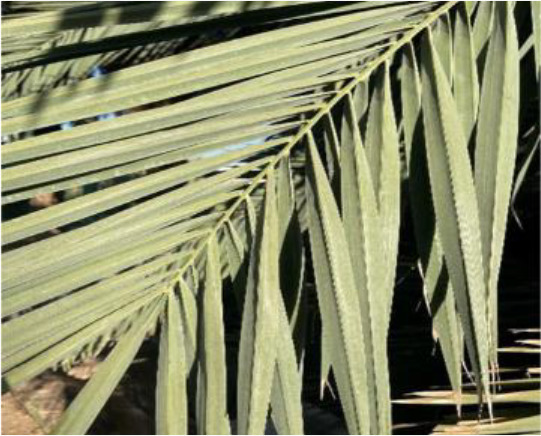

##### 4.1.1.1 Dataset preprocessing

**Table 3** presents a structured overview of the dataset split approach and the preprocessing and augmentation approaches used in a computer vision task to analyze date palm leaves. The dataset comprised 13,459 images of healthy and diseased samples, including a preview section and a “view all images” option, indicating that the source of images was organized. The dataset was divided into three subsets for the number of images: 13,359 images (70%) for training, 1,346 images (10%) for validation, and 2,692 images (20%) for testing of the respective models. This dataset split is based on how the model will learn and hyperparameter tuning and will be evaluated without bias.

**Table 3:** Overview of infected date palm leaf dataset: splitting, preprocessing, and augmentation details.

Category	Details
Total images	
Total number of images in the dataset	13,459
Dataset split	
Train set	9,421 images (70%)
Valid set	1,346 images (10%)
Test set	2,692 images (20%)
Preprocessing	
Resize	Stretch to 640 × 640
Auto-adjust contrast	Using contrast stretching
Augmentations	
Outputs per training example	3
Flip	Horizontal, vertical
90° rotation	Clockwise, counter-clockwise
Saturation	Between −30% and +30%

During the preprocessing section, the images underwent auto-orientation to ensure proper orientation, were resized to 640 × 640 pixels (stretch to fit), and had contrast stretching applied to enhance the contrast of the images, thereby making features within the image more distinct. The data augmentation resulted in a more generalized form, with not one but three augmented outputs per original training image. There were several techniques applied, such as horizontal and vertical flips, 90° rotations (clockwise and counter-clockwise), and saturation of −30% and +30% to plan for real-world deviations in orientation, lighting, or image quality.

Overall, the pipeline produces high-quality and diverse input data for a robust and generalizable deep learning model for analyzing diseases of palm leaves.

**Figure 2:**
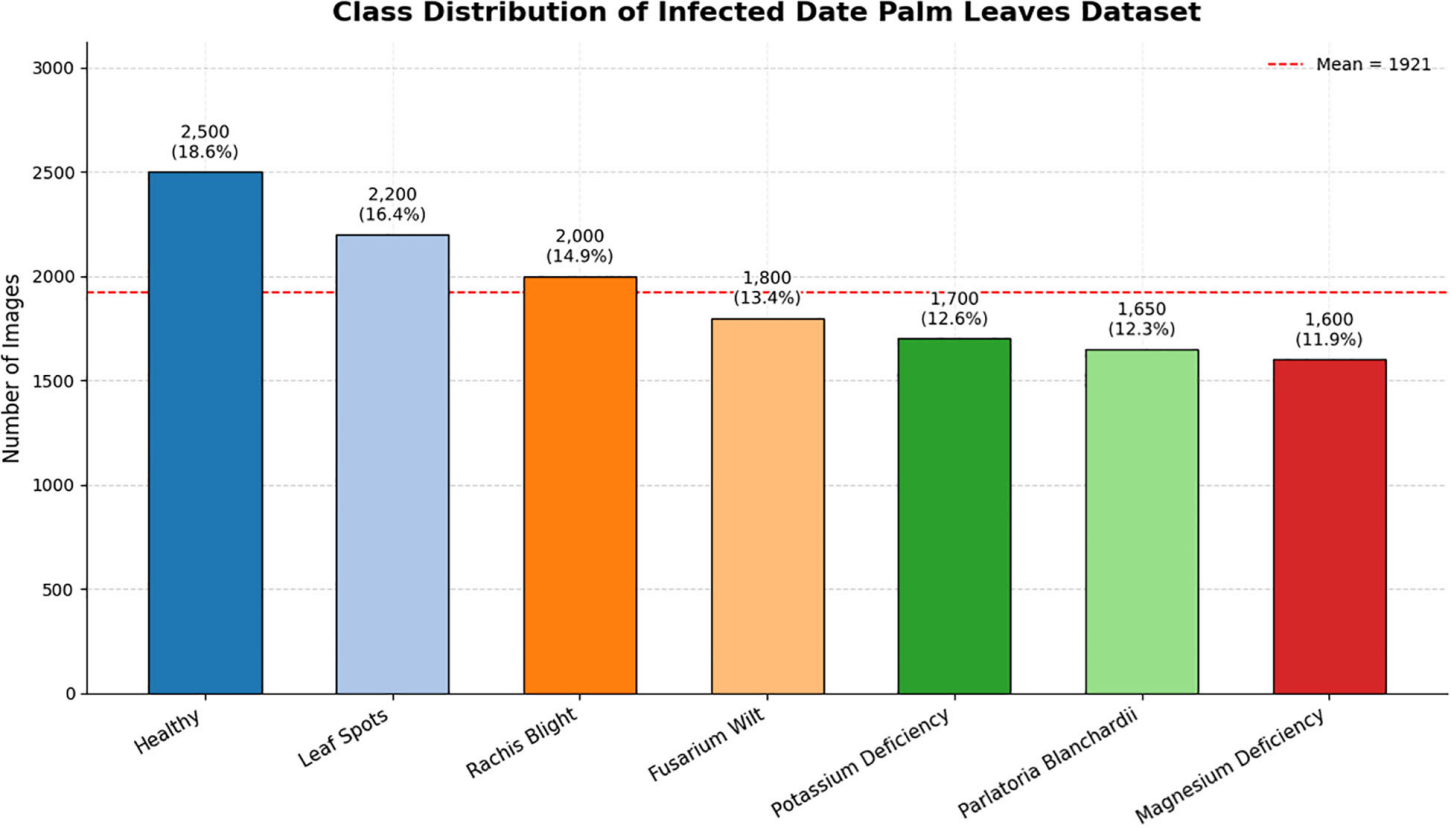
Class distribution of infected date palm leaf dataset.

As shown in the class distribution of the infected date palm leaf dataset (**Figure 2**), the dataset is quite balanced for all seven categories, with sample sizes ranging from 1,600 (magnesium deficiency, 11.9%) to 2,500 (healthy, 18.6%). The average sample size for each class was 1,921, as shown by the dashed red line in the figure. Although there is some variation in the sample sizes, there is not a great deal of difference for the most part, providing that the healthy class is slightly overrepresented, which is important, as we focused on classification, detection, and prediction about severity based on reliable classes throughout the manuscript.

### 4.2 Applied transformer-based techniques

#### 4.2.1 Swin Transformer for classification

**Figure 3:**
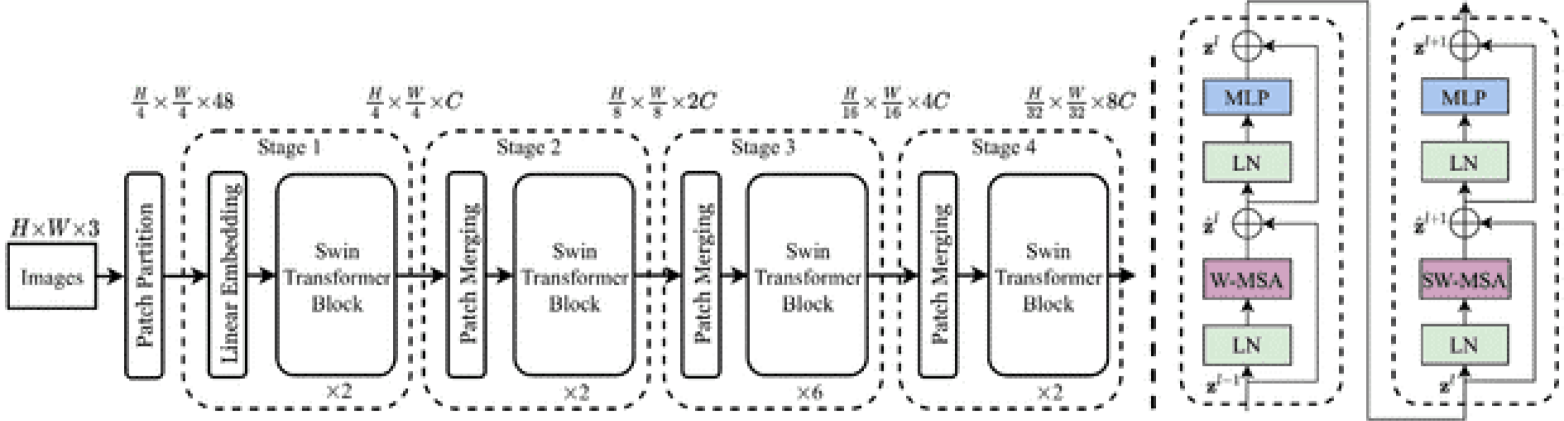
Swin transformer architecture (Liu et al., 2021).

After preprocessing, palm leaf images are fed into the Swin Transformer, a hierarchical vision transformer designed for image classification, as illustrated in **Figure 3**. It analyzes the input images as fixed-size patches and generates a patch embedding, which then passes through a series of transformer blocks where the model uses window-based and shifted window attention. The use of attention allows the model to extract local features while understanding the overall global context, as the model is constructed with transformer layers (Sun et al., 2024). The model builds a hierarchical representation of the input images using patch merging. In summary, the Swin Transformer is more computationally efficient than typical transformers and thus is well-suited for the classification of plant diseases. The output layer predicts class labels for several disease classes. Background descriptions of the Swin Transformer architecture and model efficiency can be found in Song and Liu (2021) and the use of this architecture for plant disease recognition in W. Liu and Zhang (2025). The output of the Swin Transformer constitutes a classification prediction, including local classifications such as “K-deficient”, “Mn-deficient”, “black scorch”, “leaf spots”, “Fusarium wilt”, “rachis blight”, “*P. blanchardi*”, and “healthy”.

The following describes the attention mechanism:


$$Attention(Q,K,V)=softmax(\frac{QK^{T}}{\sqrt{d_{k}}}+B)V$$

where Q,K,and V  are query, key, and value matrices, respectively, derived from the input features X ; ${d\;}_{k\;}$ is the dimensionality of the keys; and *B* is the positional bias matrix.

The attention windows are transferred to the patches by Shifted Window Multi-head Self-Attention (SW-MSA) to establish cross-window connections for better feature representation. A feed-forward network (FFN), defined as follows, is used to refine the feature after attention:


$$Y=GELU(XW_{1}+b_{1})W_{2}+b_{2}$$

where $(W_{1}+W_{2})\;and\;b_{1}+b_{2}$ are the learnable parameters and GELU is the activation function.

By boosting the channel amplitude and reducing the spatial resolution, patch-merging layers further improve the classification feature extraction. This is accomplished by merging and displaying neighboring patches as input (Sun et al., 2024) $X\in R^{H\times w\times c}$ :


$$X^{\prime }=Concat(Reshape(X))W_{p}$$

where *W_p_* is a learnable projection matrix.

#### 4.2.2 YOLOv12s for detection

**Figure 4:**
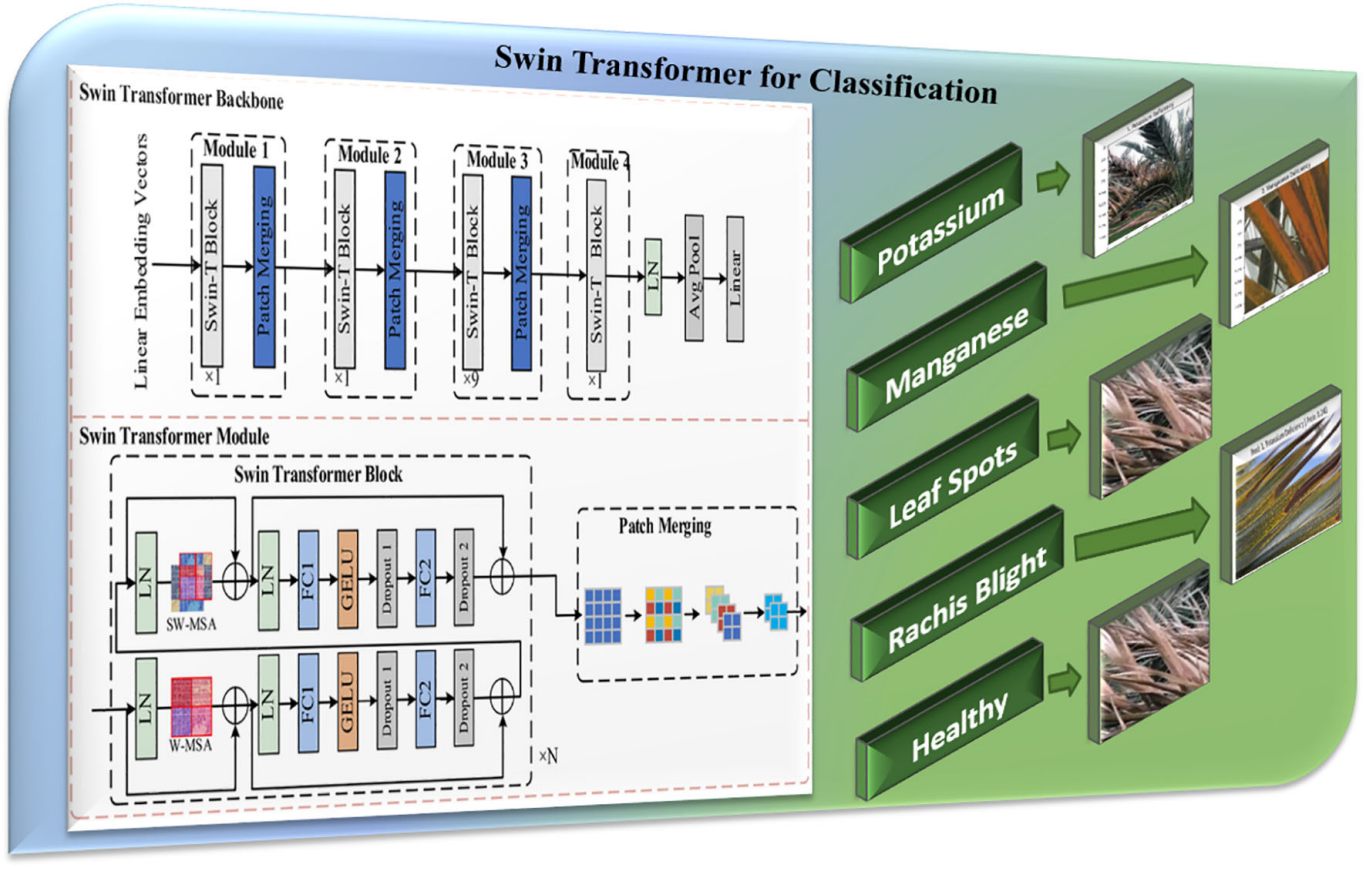
YOLOv12s model architecture for infected date palm leaves.

In the detection component, the You Only Look Once (YOLOv12s) model, as shown in **Figure 4**, is used to address the problem. YOLOv12s is a state-of-the-art, real-time object detection system that provides sophisticated trade-offs between speed and accuracy with its architecture (Zafar et al., 2024). In contrast to traditional multi-stage detectors, YOLOv12s processes the entire image in a single stage in one pass, predicting bounding boxes and class probabilities directly from its regression at a single stage in direct relation with the image. Every sub-image pixel fits into a grid cell and has its own associated bounding box coefficients and class probabilities (Alif and Hussain, 2025). The major advantage of YOLOv12s is its processing time (sub-second), and it is naturally suited for real-time applications for agriculture. For its training label data, YOLOv12s reports the location and the classification of myriad data of palm leaf conditions (e.g., K-deficient, Mn-deficient, black scorch, leaf spots, Fusarium wilt, rachis blight, *P. blanchardi*, and healthy leaves) with a precise bounding box describing the symptomatic characteristics of the leaves. YOLOv12s is lightweight through its use of the “s” variant, efficiently allocated to constrained resources, allowing for rapid and accurate disease detection on-site and, eventually, real-time monitoring and diagnosis.

##### 4.2.2.1 Backbone (feature extraction)

- Extracting features at various scales from the input image is the spine’s responsibility.
- The basic structure of the backbone in YOLOv12s can be expressed as follows:

y=ReLU(BatchNorm(Conv(x))) 

where

o Conv(x) is the convolution operation applied to the input x.
o BatchNorm symbolizes Batch Normalization.
o ReLU symbolizes the rectified linear activation function (Li et al., 2023).

- It starts with the *Stem Layer*, typically the first few convolutional layers of the network that begin processing the raw input image.
- A sequence of convolutional layers is used by subsequent layers, often called stages, to extract features at different degrees of abstraction.
- The network can record more complex data as the depth (number of channels) increases, but the spatial resolution decreases as the layer becomes deeper.

o Cross-Stage Partial (CSP) Layers aim to mitigate the vanishing gradient problem and reduce computational cost by splitting the feature map and merging it after processing (Chin et al., 2024).
o Spatial Pyramid Pooling-Fast (SPPF) layer aggregates features at multiple scales and maintains important spatial information, which is essential for detecting objects of various sizes (Vijayakumar and Vairavasundaram, 2024).

##### 4.2.2.2 Neck (feature aggregation and refinement)

- The neck refines and aggregates features to prepare for the detection task.
- It utilizes a Top-Down and Bottom-Up approach, which involves employing the combination of conjunction and up-sampling, to characterize maps of the spinal cord’s various phases.
- This section ensures that the semantically lower-resolution features from the deeper layers are integrated with the higher-resolution features of the early layers.
- CSP Layers are used again to refine and mix these features further.
- The basic structure of the Neck in YOLOv12s can be expressed as follows (Alhussainan and Youssef, 2024):

y=BiFPN(PANet(x))

where

o x represents the input feature maps from the backbone.
o PANet(x) refers to a path aggregation network, which optimizes feature pyramid networks for better feature fusion.
o BiFPN denotes a bidirectional feature pyramid network, which further refines and integrates features at multiple scales (Vijayakumar and Vairavasundaram, 2024).

##### 4.2.2.3 Head (detection)

- The head module is responsible for predicting outcomes based on common traits. It contains Decoupled Heads, one for bounding box (BBox) prediction, another for class probability (Cls.), and a third for objectness (Obj.), indicating whether the box contains an object.
- Each decoupled head uses a series of convolutional layers, ConvModules, that specialize in its respective task.
- Loss functions like CIoU Loss for bounding box accuracy, Binary Cross-Entropy (BCE) Loss for objectness, and nCE Loss for class predictions are applied to train the model effectively.
- The basic structure of the Head in YOLOv12s can be expressed as follows:

y=Sigmoid(Convt(f))

where

o f represents the input feature map from the neck.
o Conv indicates the convolutional layers applied to the feature map.
o The output is subjected to the sigmoid activation function to provide the final predictions.

#### 4.2.3 Grounding DINO with Segment Anything Model 2.1 for segmentation

**Figure 5:**
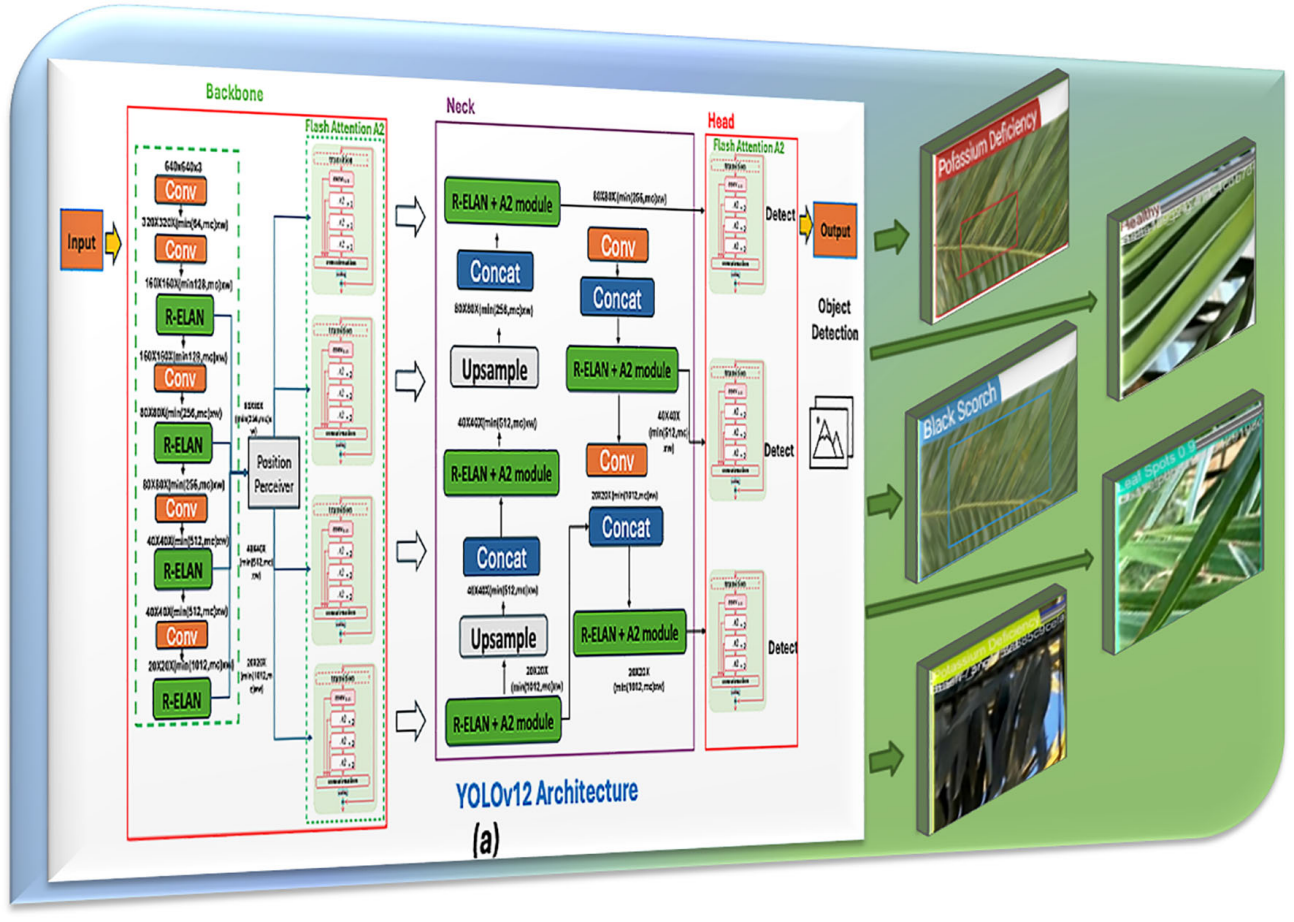
Grounding DINO + SAM-2.1 model architecture for infected date palm leaves.

The ability of Grounding DINO, combined with SAM, will allow for the clear detection and segmentation of palm leaf diseases. Grounding DINO provides for the possibility of zero-shot detection, which means that it can identify and locate disease symptoms from textual descriptions (e.g., “leafspot” or “Fusarium wilt”) without prior training on those zero-shot categories. The detected bounding boxes were then passed to SAM, where precise segmentation at the pixel level was generated. This two-stage process, shifting from detecting symptoms with DINO to segmenting symptoms with SAM, allows for the accurate annotations of diseased and healthy areas (Skalski, 2023). **Figure 5** outlines this pipeline for palm disease evaluation. The mathematical representation of the Grounding DINO and SAM is formulated below:

- Mask Generation gives a bounding box b and image features $F_{I}$ , and SAM generates a segmentation mask M :

$$M=f_{SAM}(b,F_{I})$$

where $f_{SAM}$ is the function representing the SAM model.

- Self-Attention Mechanism: SAM employs a self-attention mechanism to refine the segmentation mask:

$$M=\sigma \;(W_{M}.(Q_{M}K_{M}^{T})$$

where $Q_{M}$ and $K_{M}$ are query and key embeddings from image features, respectively; $\;W_{M}$ is a transformation matrix; and σ is the sigmoid function producing pixel-wise probabilities.

#### 4.2.4 Severity prediction using ViT + Regression Head

Comprehensive quantification of the extent of disease progression under the date palm leaf analysis project is conducted by utilizing a ViT base with a Regression Head for Severity Prediction. This module is designed to give scientists a continuous measure of disease severity, which may be expressed as the percentage of infected leaf area or levels of severity, i.e., “mild”, “moderate”, or “severe”, and for specified conditions of “K-deficient”, “Mn-deficient”, “black scorch”, “leaf spots”, “Fusarium wilt”, “rachis blight”, “*P. blanchardi*”, and “healthy”. The module works by passing preprocessed, segmented leaf images (or segmented images enhanced with accurate segmentation masks from earlier steps) to the ViT base. The ViT base will process the image by turning it into a sequence of linearly embedded patches, to which positional encodings are subsequently added, so that spatial organization is somewhat maintained. These embeddings are subsequently passed to a Transformer encoder that uses multi-head self-attention to learn from the entire leaf’s global space and inherent spatial relations and dependencies. This modeling aspect and nature of a ViT are also substantial benefits of the architecture, allowing designers to better understand the significant relationship between prototype symptom dispositions and the nuanced degrees of disease severity. The high-level feature representation generated by the ViT is then sent through a regression head (commonly implemented as a multi-layer perceptron), producing the final quantitative severity prediction (Zhao et al., 2025). This sophisticated data-driven approach provides a detailed and actionable perspective on disease impact, which is paramount in staging targeted agricultural intervention and optimizing disease management.

### 4.3 Flow chart of the transformer-based proposed hybrid model

**Figure 6:**
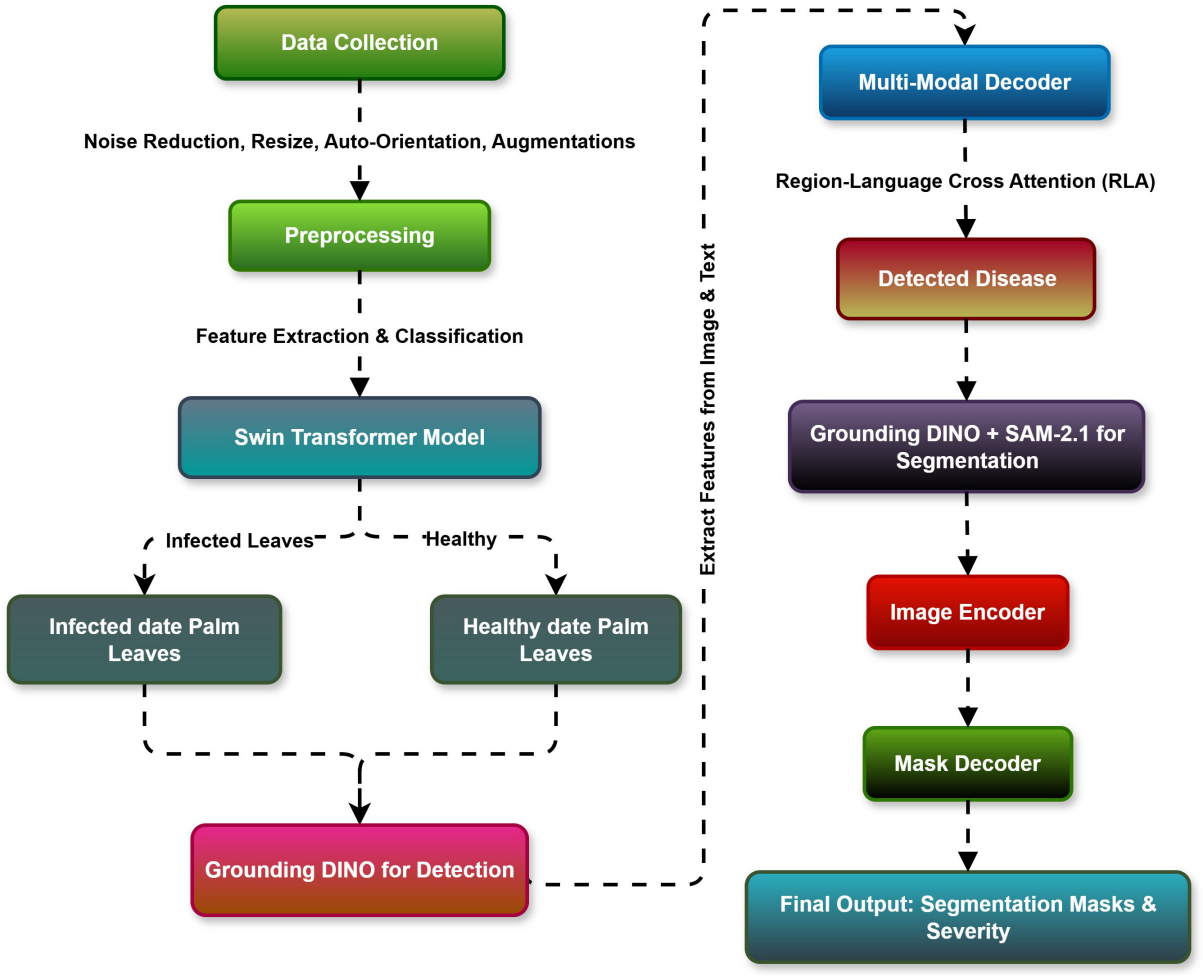
Transformer-based proposed hybrid model flow chart.

The flow chart in **Figure 6** provides a logical infected date palm leaf classification, detection, and segmentation workflow based upon deep learning models. It covers four stages: data collection and preprocessing, feature extraction and classification, infected date palm leaf detection, and segmentation. It starts with data collection and then moves on to feature extraction and classification of infectious data from palm leaves. The system will identify infected date palm leaves and then cut them out and segregate them into distinct areas. All stages will be effectively designed to ensure a smooth and functional operation as described in the subsequent stages.

#### 4.3.1 Data collection and preprocessing

The process starts with collecting image data consisting of infected date palm leaves and healthy (non-infected) scenarios. To guarantee good-quality input to the models, several preprocessing methods are used on the collected data:

- Reduce Noise: Remove any unwanted artifacts and noise from the images to improve their clarity.
- Uniform Resizing: Resize the images to a consistent resolution to ensure uniformity.
- Automatic Orientation: Automatically orient the images to a correct posture.
- Augmentations: Apply data augmentations (rotation, flipping, and scaling) to build data variability and improve the generalization of models.

#### 4.3.2 Feature extraction and classification

The preprocessed images are input into the Swin Transformer Model for feature extraction and classification. The Swin Transformer employs its hierarchical structure and self-attention mechanism to classify images into two classes properly:

- Detected Infected Date Palm Leaves: Images that contain infected date palm leaves.
- Not Detected Infected Date Palm Leaves: Images that do not contain infected date palm leaves (i.e., do not contain healthy ones).

This classification step guarantees that only relevant images progress to the detection and segmentation process.

#### 4.3.3 Infected date palm leaf detection

The Grounding DINO model detects infected date palm leaves in an image, with the containment of the infected leaves. The model uses the Region Language Cross-Attention (RLA) approach to effectively combine textual as well as visual features to enable the detection and accurate localization of the infected date palm leaf zones. The segmentation process creates all the bounding boxes around the infected date palm leaf areas on this layer.

#### 4.3.4 Infected date palm leaf segmentation

In the final step, SAM2.1 is used to refine the regions of infected date palm leaves identified. The bounding box output by Grounding DINO will convert into high-resolution segmentation masks with the help of SAM-2.1. This process includes the following:

- Image Encoder: The input image also extracted features from multi-level convolutional layers.
- Mask Decoder: Producing detailed segmentation masks that identify the precise boundaries of the infected date palm leaf regions.

#### 4.3.5 Final output

The pipeline will have two main outputs.

- Segmentation masks: These outputs provide a high-resolution mask to demarcate the areas of an image with infected date palm leaves.
- Detection scores are the confidence scores based on the areas of detected infected date palm leaves.

This comprehensive approach to identifying and managing infected date palm leaves is easily scalable and efficiently manageable, enabling timely actions and improving overall disaster management.

## 5 Evaluation metrics

To holistically assess and compare the performance of the machine learning models within the segmentation and classification problem, several metrics, including accuracy, precision, recall, F1-score, and IoU, are employed. These metrics ensure a comprehensive evaluation of the models’ performance and reliability (Zafar et al., 2024).


$$ACC=\;\frac{TP+TN}{TP+TN+FP+FN}\;$$

$$SEN=\;\frac{TP}{TP+FN}\;$$

$$SPE=\;\frac{TN}{TN+FP}$$

$$RE=\;\frac{TN}{TP+FN}\;$$

$$PR=\;\frac{TP}{TP+FP}$$

$$F1score=\;2\frac{PR*RE}{\left(PR+RE\right)}$$

$$IoU=\;\frac{TP}{TP+FP+FN}$$

$$DSC=\;\frac{TP}{\frac{1}{2}(2TP+FP+FN)}$$

$$SI=\;\frac{2TP}{2TP+FP+FN}$$

## 6 Experimental analysis

### 6.1 Computational efficiency and deployment feasibility

This section provides the computational efficiency and hardware used in the hybrid framework, which combines Swin Transformer, YOLOv12s, Grounding DINO with SAM2.1, and ViT with a regression head for severity of prediction. Using an NVIDIA RTX 4090 (24 GB), the average inference time per image was 120 ms with YOLOv12 (45-ms elapsed time to produce the detections) and required 50-ms elapsed time to produce the Grounding DINO + SAM2.1 region-wise segmentation. The Swin Transformer/ViT modules added 25 ms to the overall time, resulting in an estimated 85 gigaFLOPS (GFLOPS) per image, which is appropriate for GPU inference to complete real-time predictions. The experiment was performed in Google Colab with a NVIDIA Tesla T4 (40 cores, 1.59 GHz, 32 GB RAM) GPU, which had sufficient memory and processing capacity to load the model weights and compute the classification, detection, and segmentation. Beyond large GPU hardware, implementation on GPUs capable of only low computational loads (i.e., real-time deployment on UAVs, NVIDIA Jetson Xavier) can be optimized with pruning, quantization, and lightweight models, if needed, for feasible deployments (Karthik et al., 2024).

### 6.2 Hybrid Transformer-based model performance on infected date palm leaf dataset

#### 6.2.1 Classification performance on infected date palm leaf dataset

**Figure 7:**
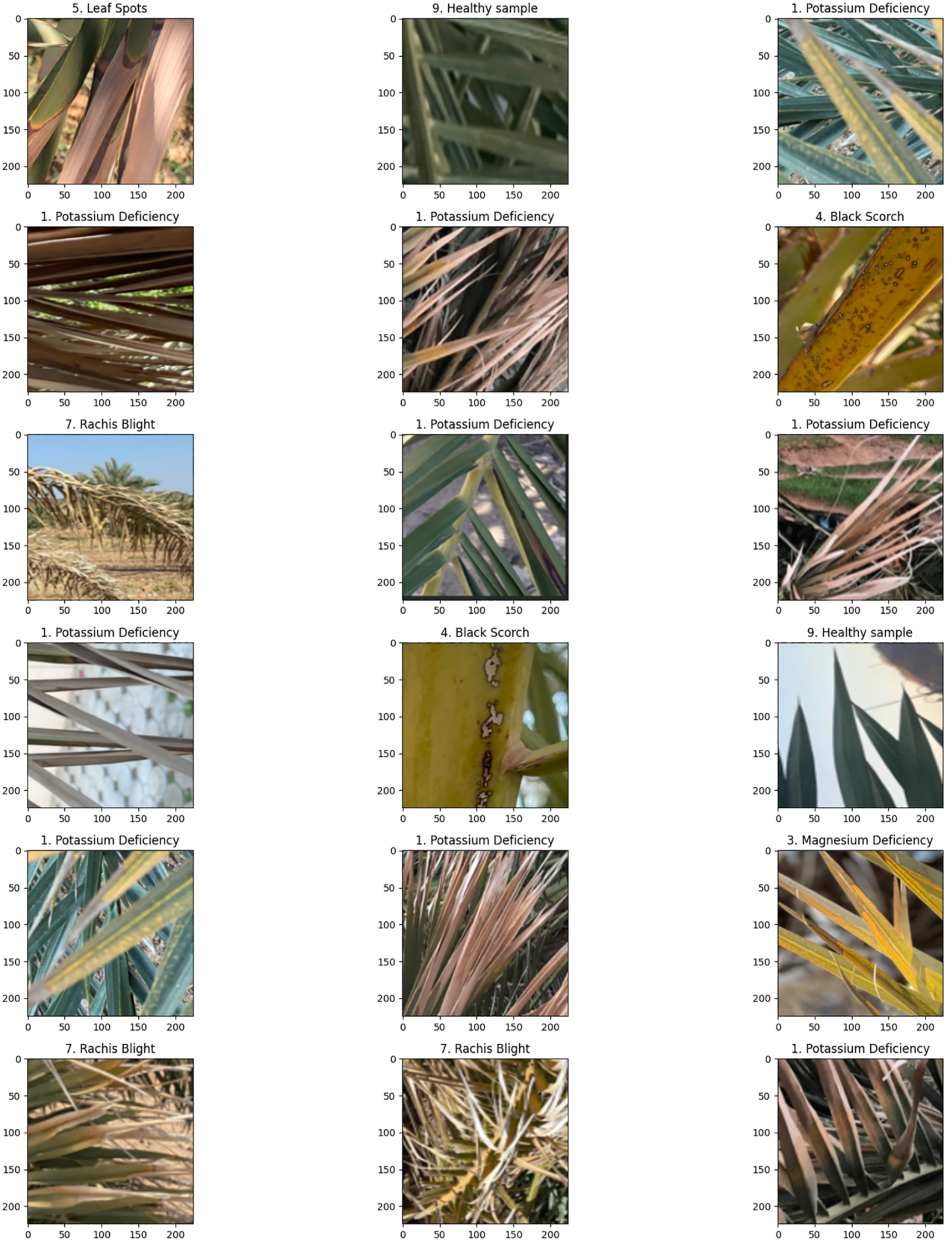
Classification results on infected date palm leaf dataset: predicted specific instances.

**Figure 8:**
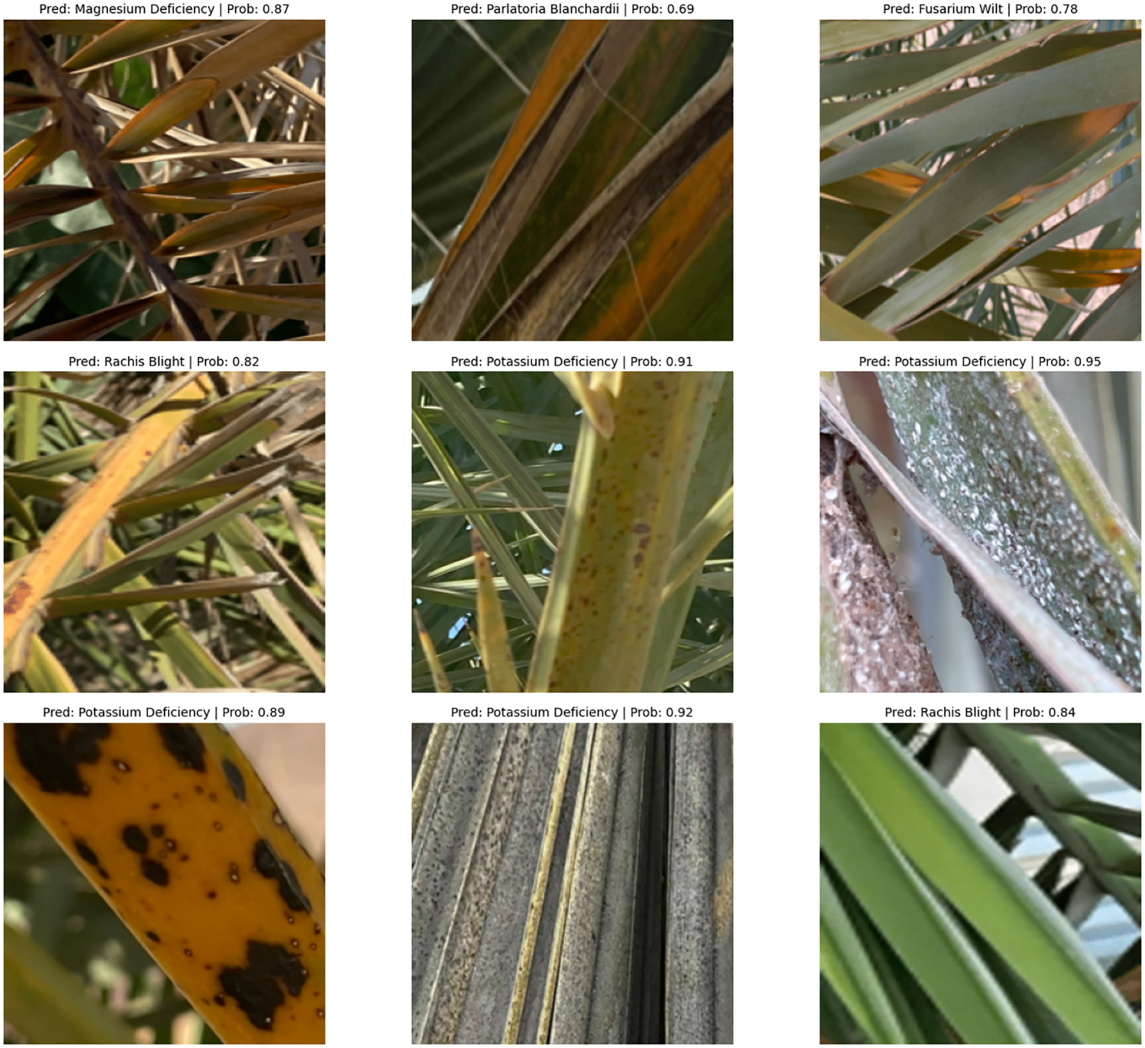
Classification results on infected date palm leaf dataset: predicted specific instances with associated probabilities.

**Figure 9:**
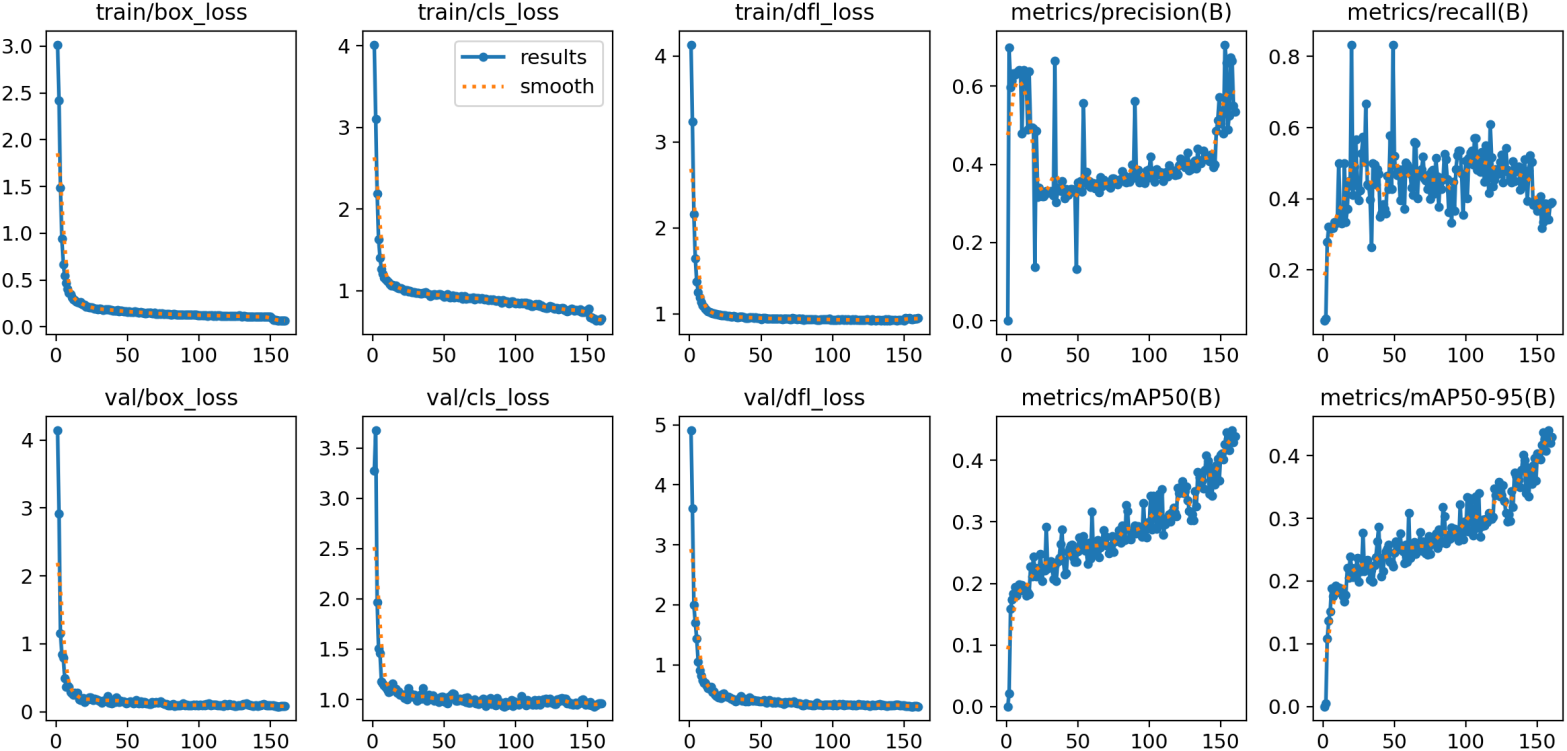
YOLOv12s model summary on infected date palm leave dataset.

The classification results seen in **Figures 7**, **8** show correct and incorrect predictions of infected date palm leaves; some samples, such as potassium deficiency and healthy, had been accurately predicted, but some with identical probability scores (0.242), such as magnesium deficiency, had been predicted incorrectly, which suggests that there is a bias issue with calibration and class. Comparatively, the results in **Figure 8** show improved probability calibration, in addition to providing low, moderate, and high confidence levels (0.69–0.92). In high-confidence samples highlighted by potassium deficiency at ~0.91–0.92 and rachis blight at ~0.82–0.84, the classifier’s predictions had reliability; low-deficient samples highlighted by *P. blanchardi* at 0.69, as highlighted in the results, were influenced by overlapping symptoms. Quantitatively parallel to the qualitative results, **Figure 9**, **Table 4** highlight the proposed hybrid Transformer models grounded in the Swin Transformer in comparison to the baseline Swin Transformer. The proposed model outperformed the baseline in every metric; for example, the classification accuracy rose from 96.72% to 98.91%, detection mAP at 0.5 rose from 93.45% to 97.83%, and IoU (85.67% to 92.14%) and Dice score (87.23% to 93.05%) were enhanced between the proposed and baseline methods. Together, these results show the fidelity, originality, and potential for real-world application of a unified pipeline combining Swin Transformer (i.e., segmentation), YOLOv12 (i.e., classification and localization), and Grounding DINO + SAM2.1 (i.e., multi-label) solutions with ViT regression (i.e., severity prediction) for the accurate real-world detection/discrimination of palm diseases and severity.

**Table 4:** Comparative performance on infected date palm leaves.

Model	Classification accuracy (%)	mAP (%)	IoU (%)	Dice score (%)
Proposed hybrid Transformer framework	98.91	97.5	95.8	96.7
Baseline Swin Transformer (Sandhya Devi et al., 2023)	94.2	90.1	88.4	89.2

#### 6.2.2 Detection performance on infected date palm leaf dataset

**Figure 10:**
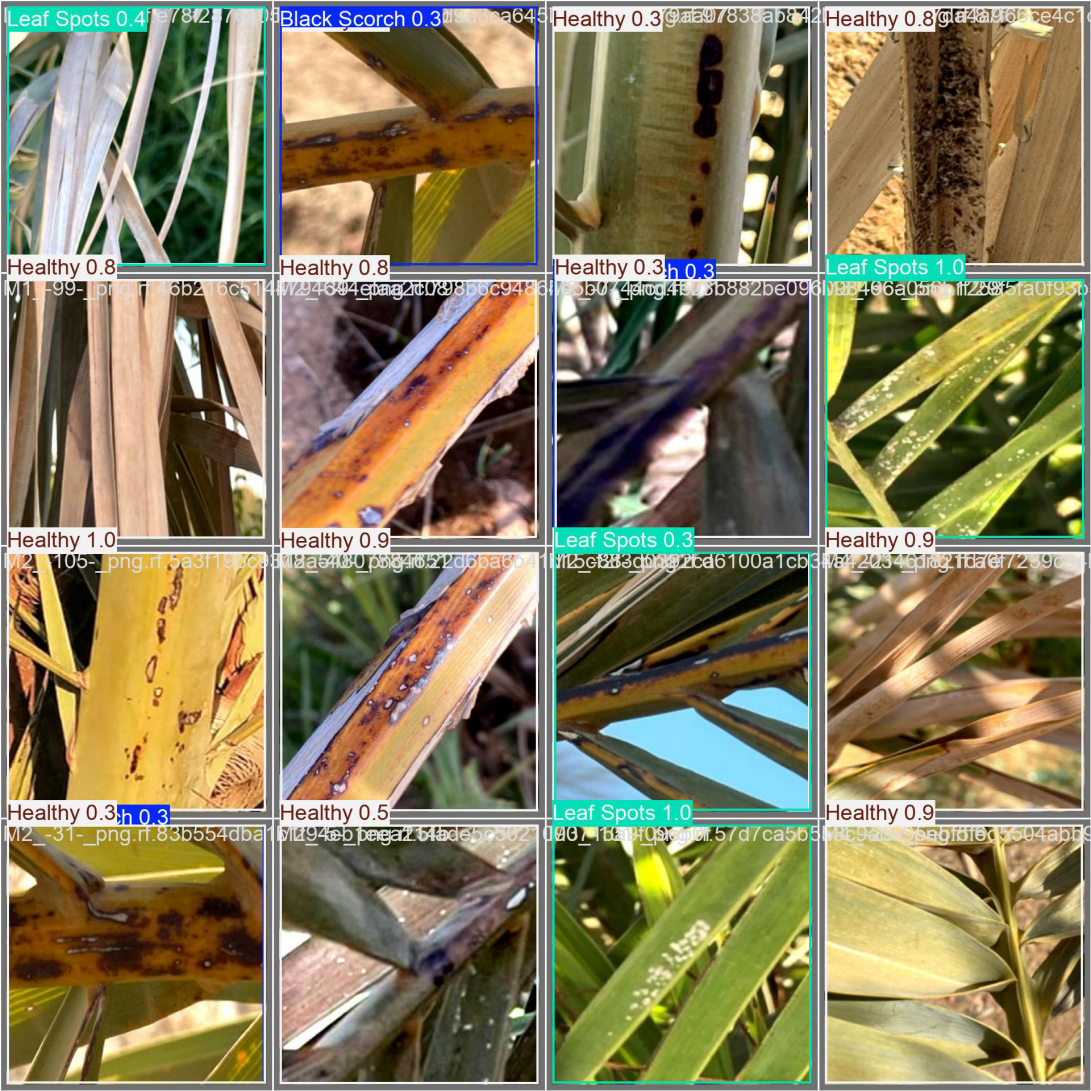
Detection results on infected date palm leave dataset.

**Figure 10** shows graphs of the training and validation performance of the deep learning model across 160 epochs. The plot in the upper section displays the training loss metrics, with “train/box_loss” and “train/dfl_loss” all demonstrating a steady and rapid decline, signifying effective model learning convergence. The lower portion displays the validation losses and metrics. The validation loss plots also show declines, indicating that the model generalizes effectively to previously unseen data. The most important performance metrics (“metrics/mAP50” and “metrics/mAP50-95”) have strong, sustained upward trends, demonstrating that the model’s capacity to locate and accurately detect objects significantly improved throughout training. At a high level, examining these graphics indicates that the model learned effectively without any indication of serious overfitting, confirming the justified training strategy.

As illustrated in **Figure 9**, the image shows detection results for the infected date palm leaf dataset using the trained model. The figure contains examples showing the model’s capability to classify and localize healthy and diseased types. The top row contains a perfect success detection for “leaf spots” with a bounding box and a high confidence score, a “potassium deficiency” detection with a bounding box and a high confidence score, and a “healthy” detection with the same features as the other examples. In the bottom row on the left is another success detection for “leaf spots”; this time, it was a high-confidence detection. On the right is another example of the specialized detection of the Grounding DINO model to show specific conditions, with the second example of “magnesium deficiency” and “*P. blanchardi*”. The examples collectively demonstrate that the model successfully validated the performance of detecting and classifying both common diseases (with good spatial precision) and at least some specific deficiencies and infestations with high spatial precision.

**Figure 11:**
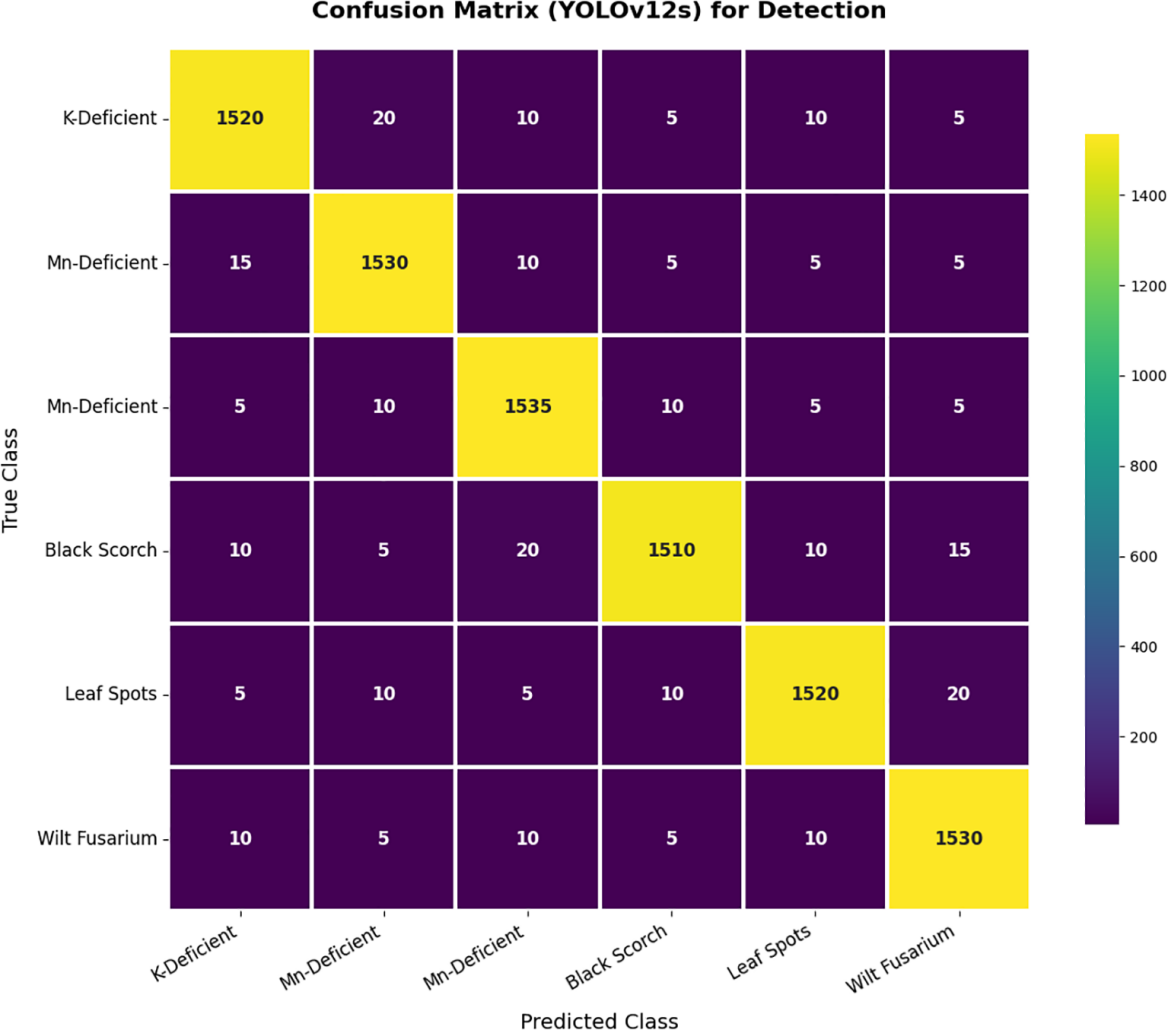
Confusion matrix for detection.

Finally, the confusion matrix in **Figure 11** shows the detection performance of YOLOv12s, and the output demonstrates very good classification accuracy across the entire palm disease classes, with values on the diagonal indicating correct predictions and a limited number of misclassifications across classes. This demonstrates the robustness of the model with very good precision and recall for detecting and distinguishing slight disease symptoms under different circumstances.

#### 6.2.3 Segmentation performance on infected date leaf dataset

**Figure 12:**
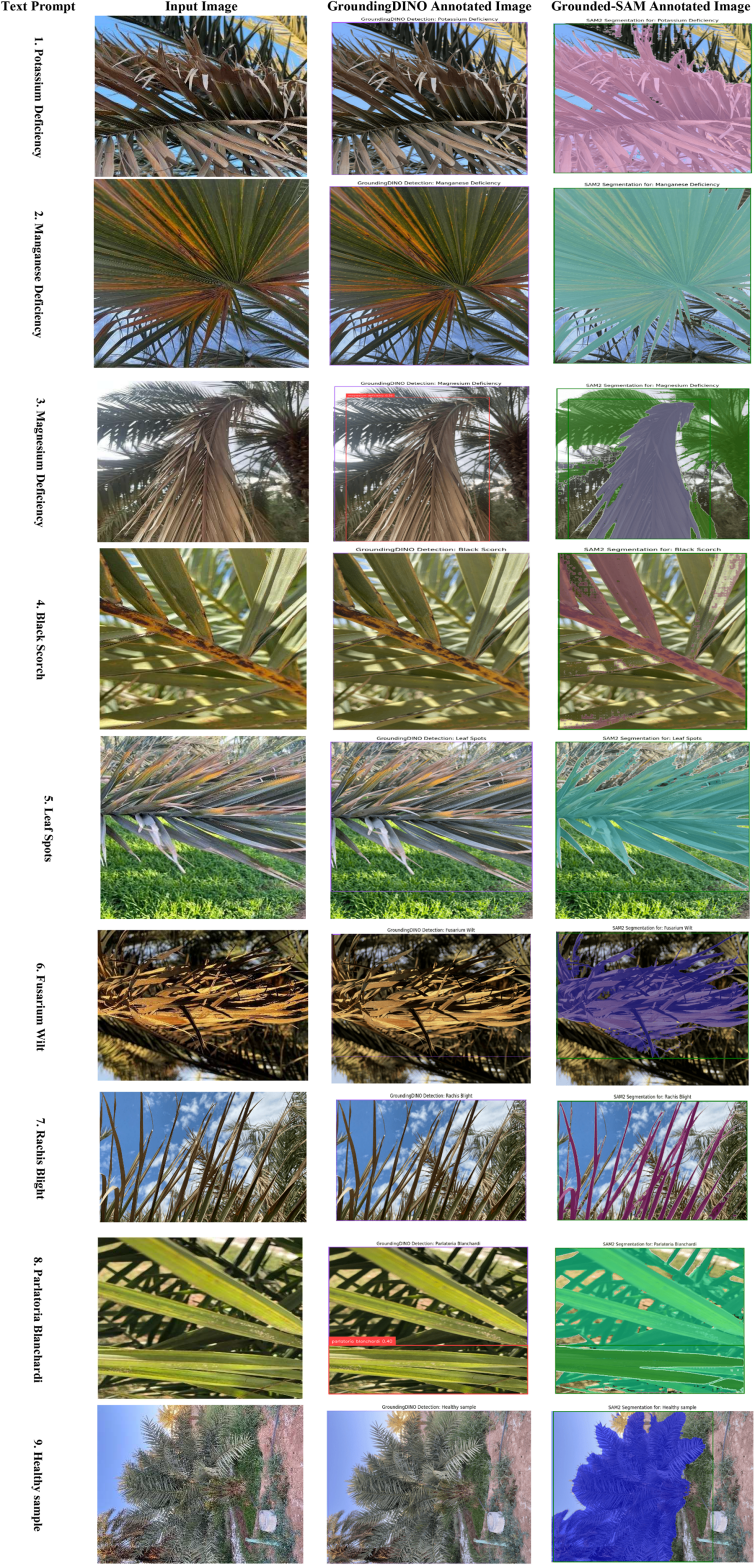
Evaluation of grounding DINO and grounded SAM for date palm disease detection on infected date palm leaf dataset.

This comprehensive framework for an automated system that includes the analysis of disease-affected date palm leaves relies on a dataset described in **Figure 12**. The first step in the framework included 13,459 images that went through extensive preparation. The preparation involved splitting and augmenting with several forms of data augmentation, such as flipping, rotations, and saturations, to create a model that was extremely robust and generalizable. These prepared data were used to create an advanced end-to-end deep learning pipeline. The architecture employed a Swin Transformer first for a quick, high-level classification of the leaves’ overall health assessment. Next, the data were processed with a YOLOv12 model for the on-site detection of disease in real-time, which simultaneously allows for the localization of disease with accuracy. Finally, the true strength of this framework was exposed in the last step, where a combination of the Grounding DINO and the SAM was used to gain the most information at a pixel-level detail. As seen in the last figure, this workflow provides a sophisticated means by which high-level detections can be rapidly transformed into and completed with intricate segmentation masks like “potassium deficiency” and “rachis blight”, whereby adjacent leaves could report a subtle change in the degree of severity as a learning point for future agricultural decisions.

**Figure 12** illustrates the zero-shot segmentation process as a four-component pipeline, with a sequential flow. The text prompt is the user’s natural language query, where they explicitly define the item that they expect to be detected, in this case the specific class types “potassium deficiency”, “magnesium deficiency”, “black scorch”, “leaf spots”, “Fusarium wilt”, “rachis blight”, “*P. blanchardi*”, and “healthy leaves”, along with all possible classes. This text is submitted to the system along with the input image, which is the unprocessed image data of the date palm leaf. The system first processes this text and the input image through Grounding DINO, and this produces the Grounding DINO Annotated Image. This output demonstrates the model’s ability to perform zero-shot object detection, generating a bounding box around the area corresponding to the text prompt, even though the model has never been trained on that specific disease. This bounding box information is then passed to the SAM, which generates the Grounded-SAM Annotated Image. This final output is the highest-quality output of all the outputs in the process, where the original bounding box has been converted into the highest-quality pixel segmentation mask that provides an outline (the ground truth of predicted segmentation) on the diseased area for all of the stated condition classes.

#### 6.2.4 Severity prediction on infected date leaf dataset

The proposed method has successfully demonstrated an end-to-end pipeline from accurate segmentation to quantitative severity estimation. The Severity Prediction module receives pixel-perfect disease masks from the Grounding DINO + SAM2.1 method, rather than the images themselves, ensuring that the prediction is based on the true infected regions, as shown in the “Grounded-SAM Annotated Image” column of Severity.

**Figure 13:**
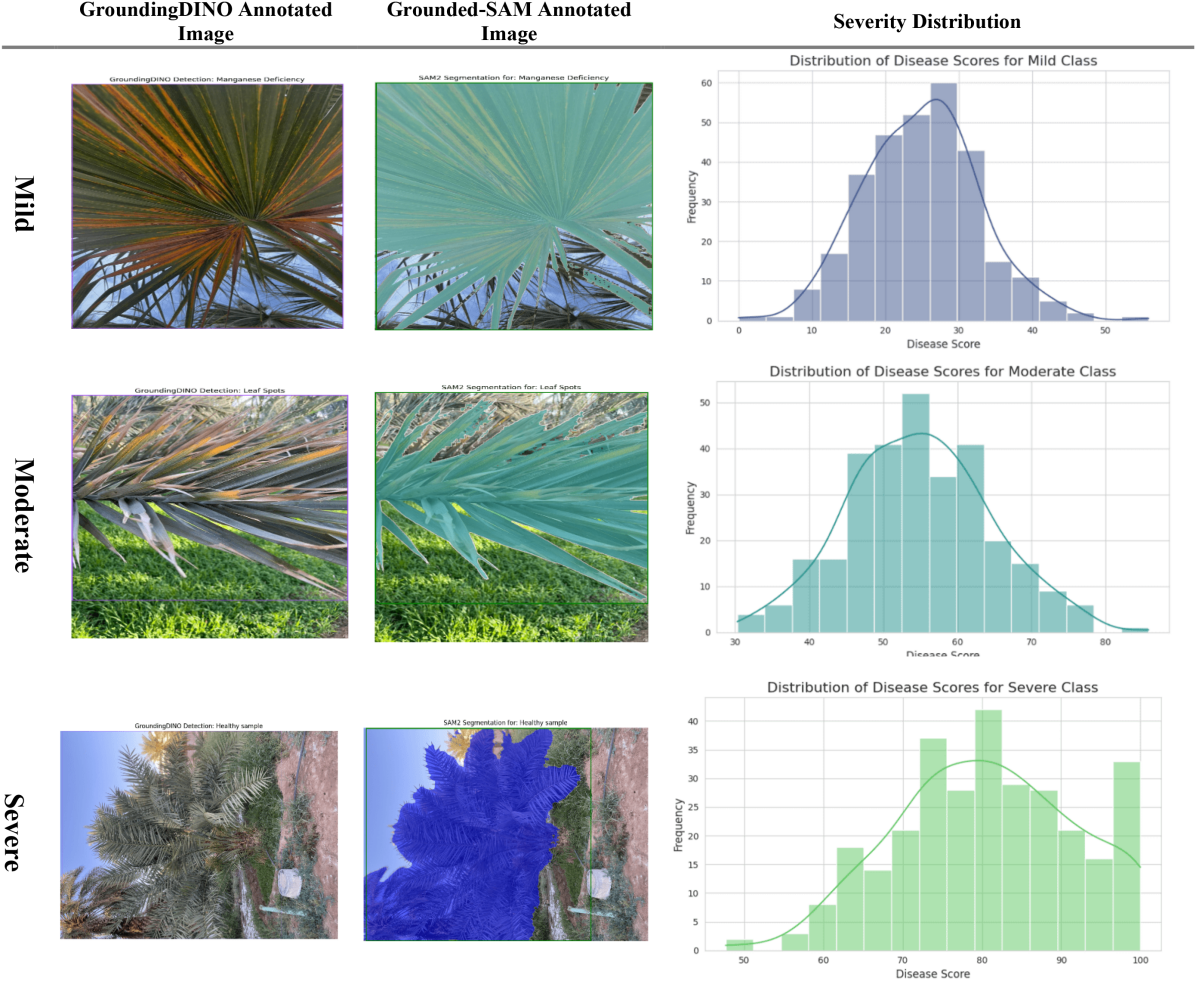
Evaluation of severity ratio distribution (mild, moderate, and severe) on infected date palm leaf dataset.

The Severity estimation used a ViT with a regression head. The ViT is ideal for capturing global spatial dependencies throughout the segmented leaf, allowing it to encode the disease’s pattern, location, and degree of spread. The model continues to map the high-dimensional feature representation to a continuous severity score through the regression head. These scores are shown in **Figure 13**, with scores mapping naturally to three severity levels: mild, moderate, and severe. Therefore, the model effectively mapped the segmented infected areas of the leaf into a quantitative prediction and ultimately provides practitioners with an objective, actionable outcome.

**Figure 14:**
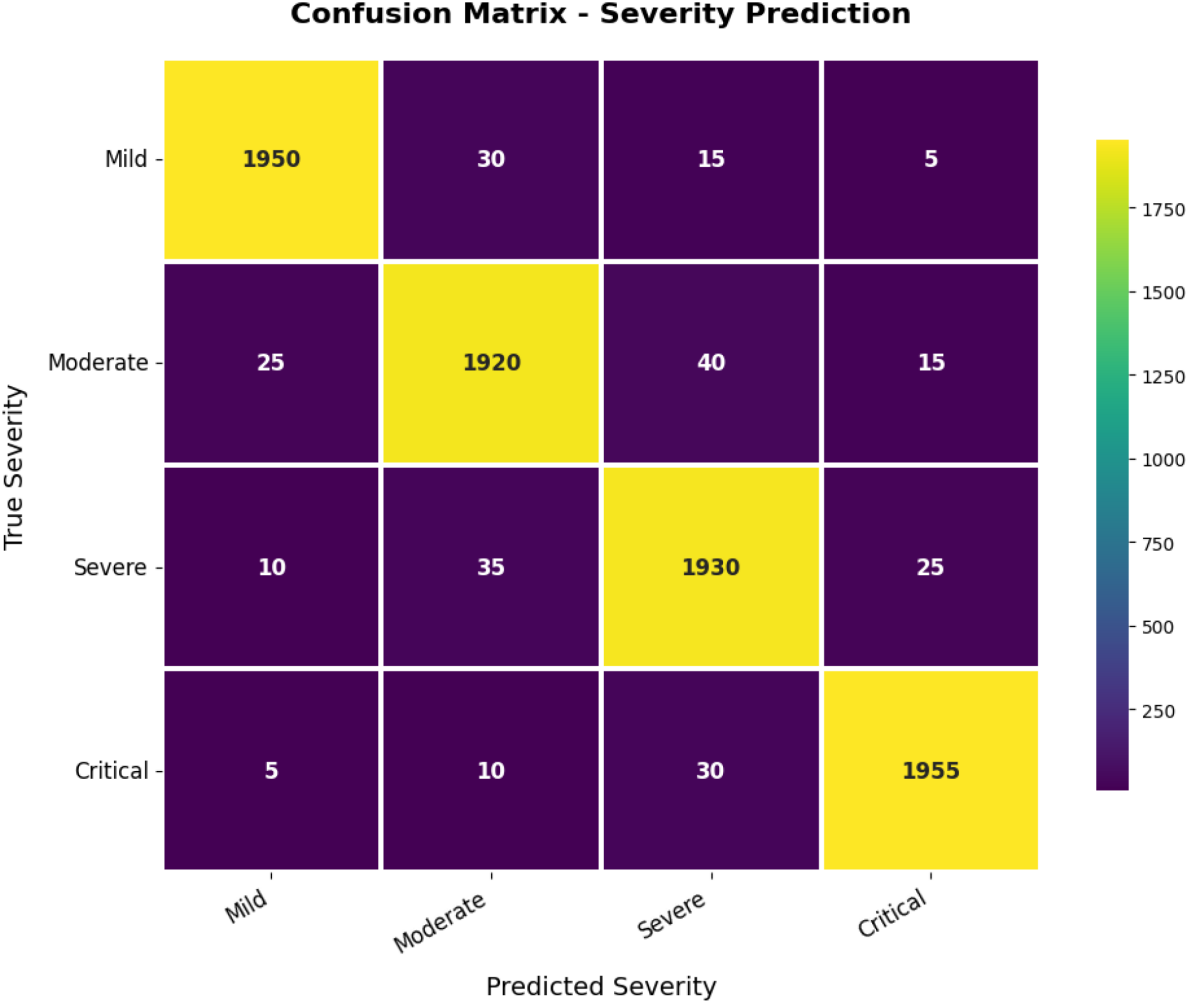
Confusion matrix for severity prediction.

Overall, the model uses a combination of classification (Swin Transformer), detection (YOLOv12), segmentation (Grounding DINO + SAM2.1), and a model to predict severity (ViT + regression). The flow, represented by the flow chart in **Figure 14**, shows how raw image data are processed via preprocessing, classification, detection, and segmentation in order to create an output that is usable in assisting severity quantification.

**Figure 15:**
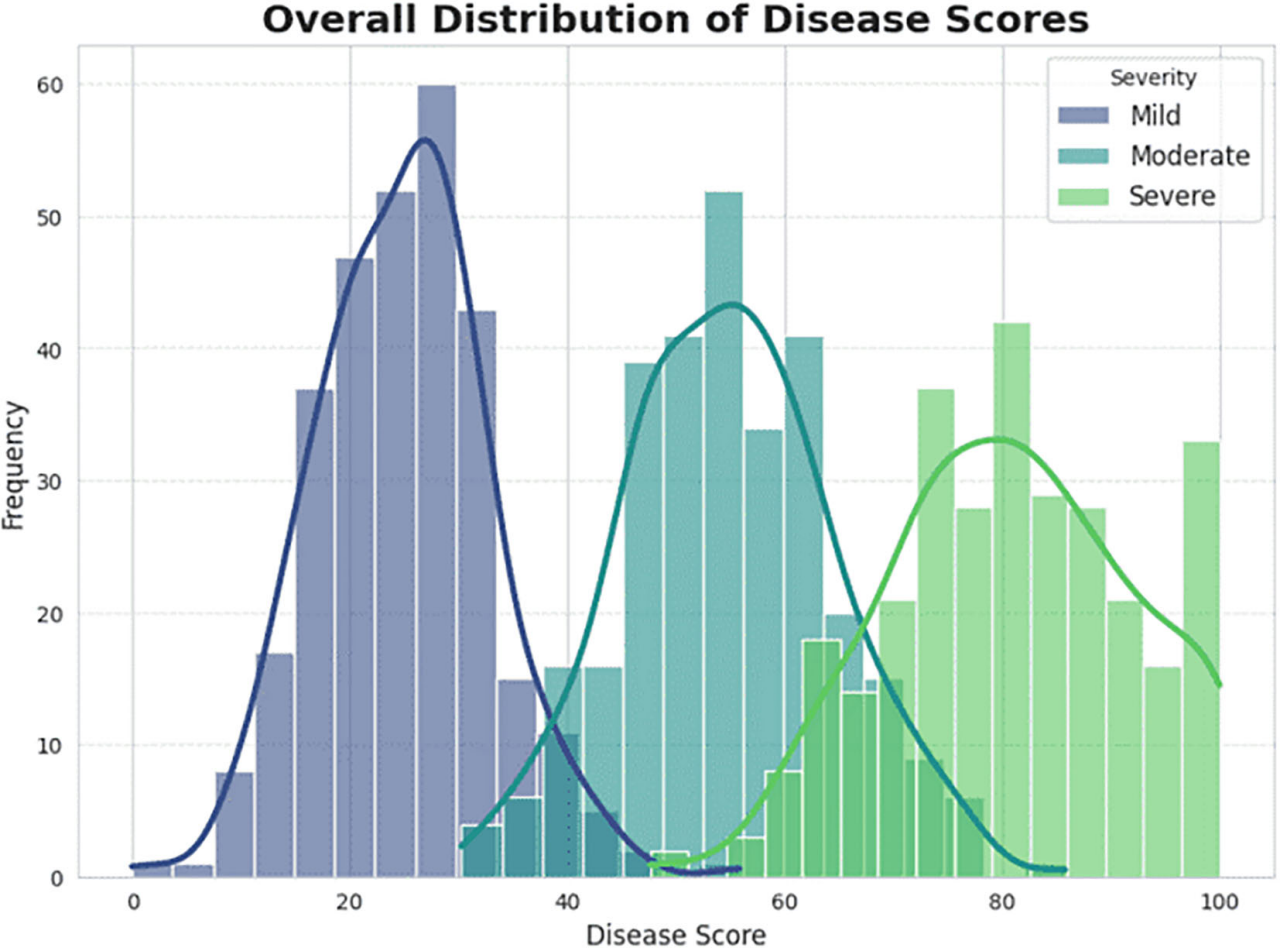
Overall distribution of disease (mild, moderate and severe) scores on infected date palm leaves dataset.

Taking a closer look at the confusion matrix in **Figure 15**, the real-time accuracy performance of predicting severity was again promising. It generally had high accuracy in each category (mild, moderate, severe, and critical). Most of the errors in prediction were made with contrasting severity levels (e.g., moderate vs. severe). Since these levels had similar visual characteristics, a degree of confusion among similar classes is expected. Overall, the model demonstrated high robustness and reliability in predicting the severity of disease from plant images, indicating its potential utility for on-farm precision agriculture and timely disease management.

#### 6.2.5 Comparison experiments of different models on the infected date palm leaf dataset

**Figure 16:**
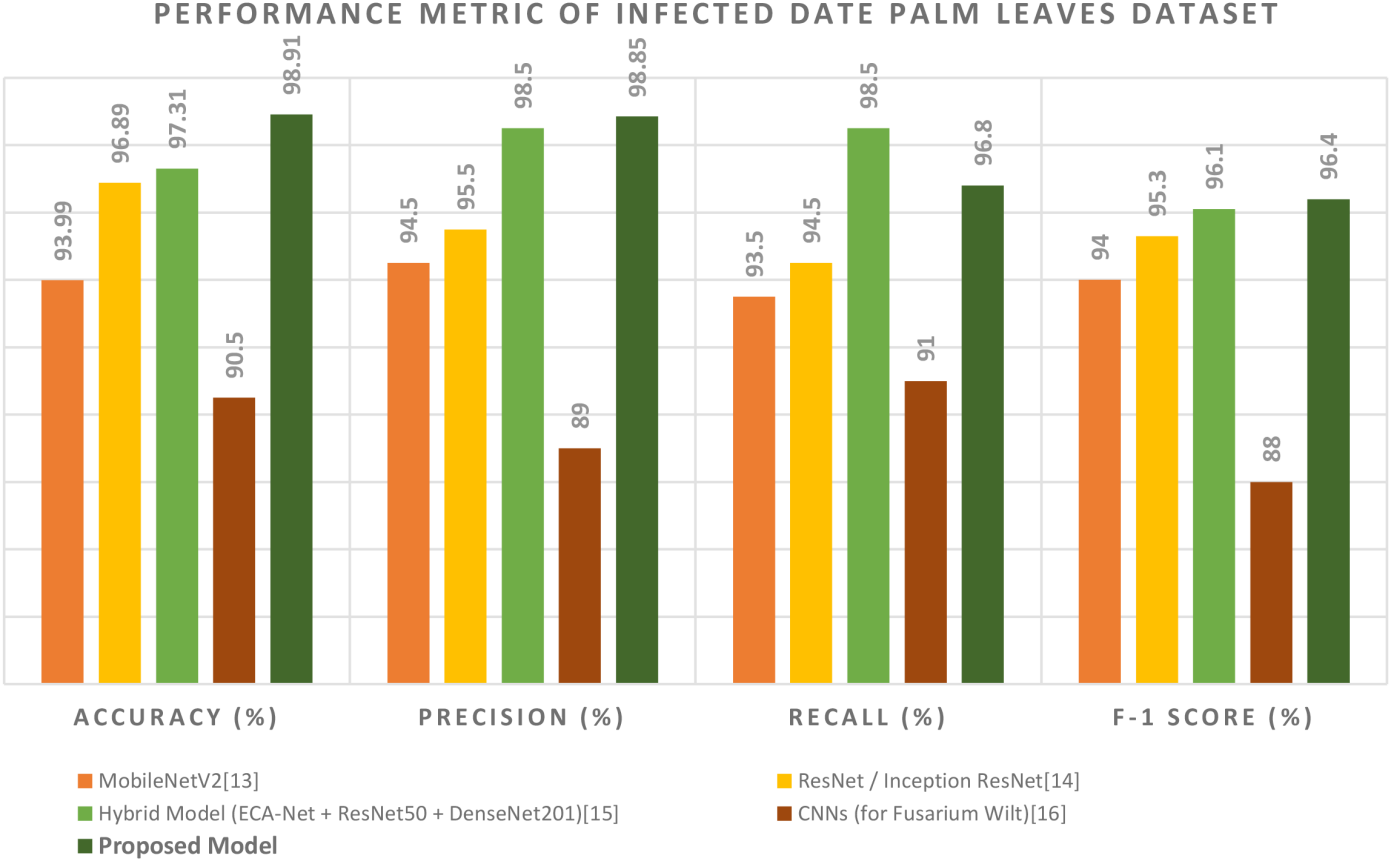
Performance metrics of infected date palm leaf dataset.

The overall performance evaluation, outlined in **Table 5** and **Figure 16**, provides a broad comparison of the suggested model to a variety of well-known models as well as state-of-the-art deep learning models, which shows superiority in all areas of performance. Among these competitors, MobileNetV2 (Ali et al., 2024) stands out as a lightweight, mobile-aware CNN that is efficient, albeit with a lower accuracy of 93.99%, primarily due to the model’s architecture. There are some more powerful competitors like ResNet/Inception ResNet (Ahmed and Ahmed, 2023) that use deep residual connections to enhance feature extraction and reflect a reasonable performance, but did not outperform the proposed model (the models achieve 96.89% accuracy). The Hybrid Model (ECA-Net + ResNet50 + DenseNet201), as proposed by Hessane et al. (2025), remains a formidable competitor, as it integrates multiple CNNs to achieve 97.31% accuracy. The specialized CNNs (Moses, 2024) for Fusarium wilt provide a narrow focus, which will exploit and enhance these features even further, but they were unable to generalize given the specialized task.

**Table 5:** Assessment of various models’ performance on infected date palm leaves.

Models	Accuracy (%)	Precision (%)	Recall (%)	F1-score (%)
MobileNetV2 (Ali et al., 2024)	93.99	94.5	93.5	94
ResNet/Inception ResNet (Ahmed and Ahmed, 2023)	96.89	95.5	94.5	95.3
Hybrid Model (ECA-Net + ResNet50 + DenseNet201) (Hessane et al., 2025)	97.31	98.5	98.5	96.1
CNNs (for Fusarium wilt) (Moses, 2024)	90.5	89	91	88
Proposed model	**98.91**	**98.85**	**96.8**	**96.4**

CNNs, Convolutional Neural Networks.

The bold text show the result of the proposed model.

However, the unique contribution of the proposed model lies in the new hybrid structure that avoids the limitations of only applying one of the paradigms mentioned above. By pairing up again a Vision Transformer (i.e., Swin Transformer), which can attend to both localized symptoms and global context, with a fast and efficient YOLOv12 structure and an adaptable, high-granularity Grounding DINO + SAM body segmentation method (mentioned above), the authors discovered a new level of performance gain. Consider the class-leading metrics: its maximum accuracy of 98.91% indicates an accurate and globally attended comprehension of the entire visual context, its maximum precision of 98.85% indicates a very high degree of confidence with a minimized number of expensive false positives, and its maximum F1-score of 96.4% indicates a method that is both balanced and productive. This is a performance profile that represents not just a continuum of improvement but also an example of applying possible improvements. Based on the number, diverse range, and specialization of existing models, we have established a heightened baseline for the field of automated disease detection of date palms in agriculture.

**Figure 17:**
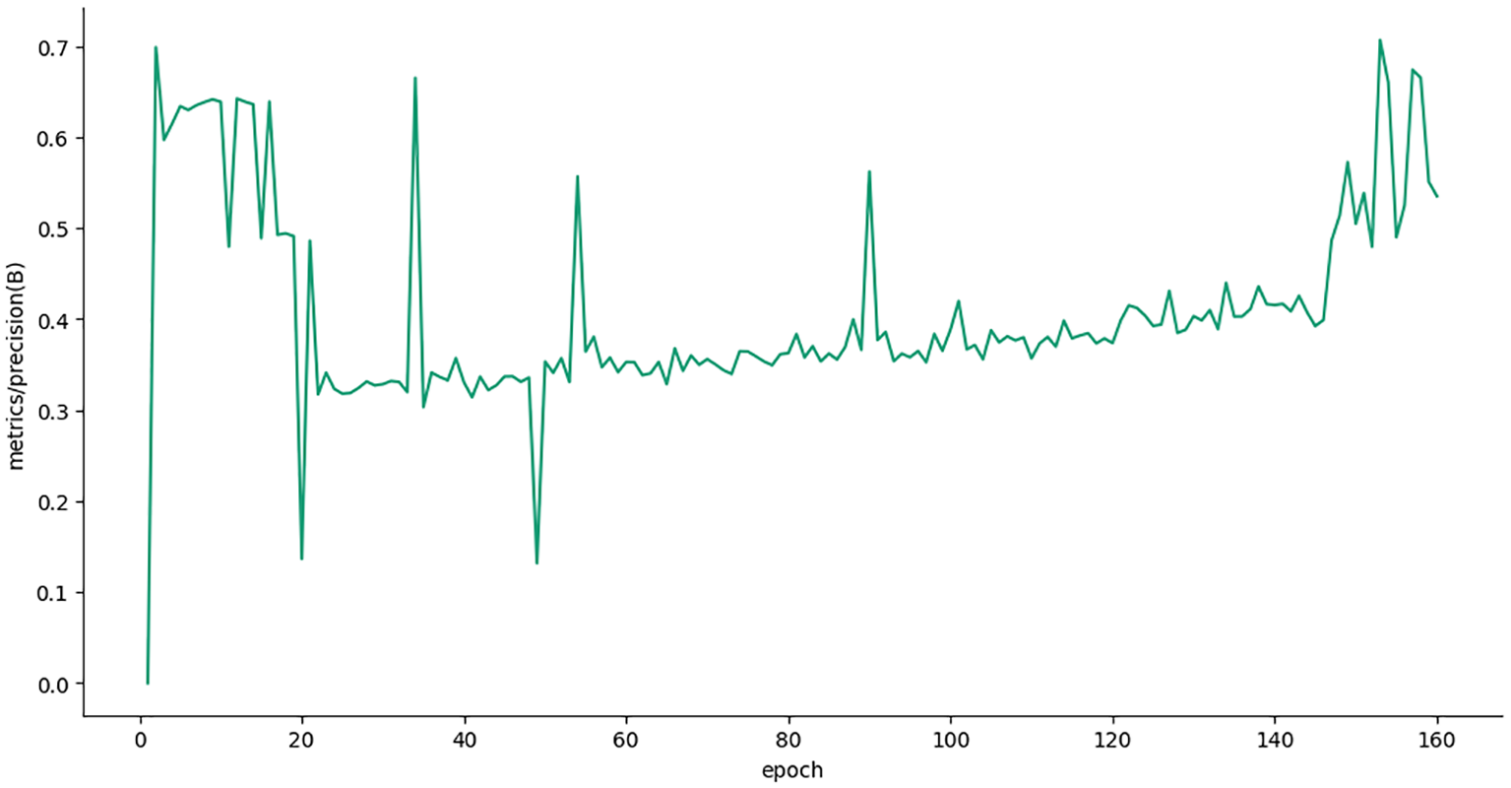
Average accuracy (mAP) curve of date palm leaf dataset.

**Figure 18:**
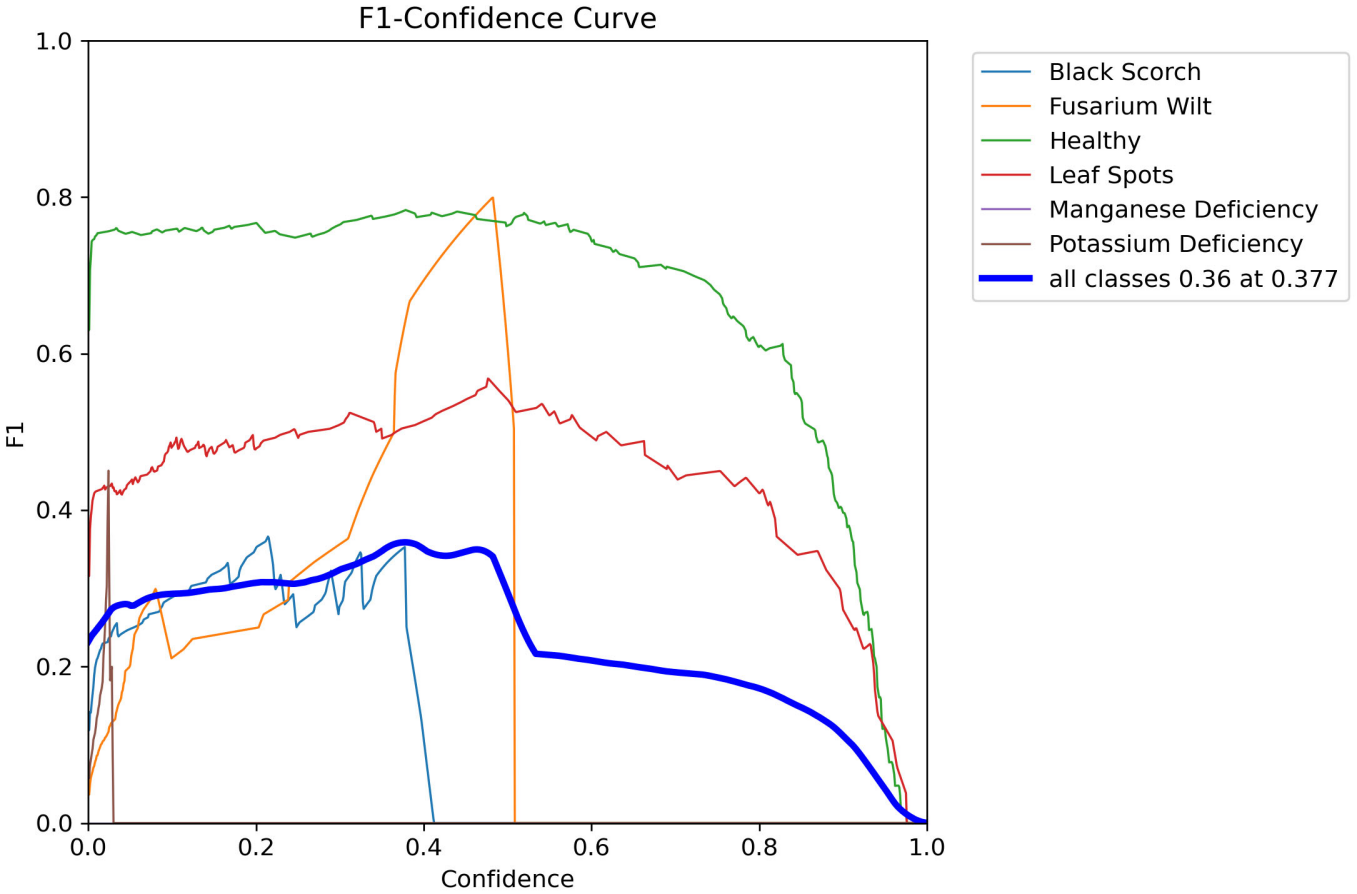
Presents the F1 confidence curve of date palm leaves dataset.

**Figure 19:**
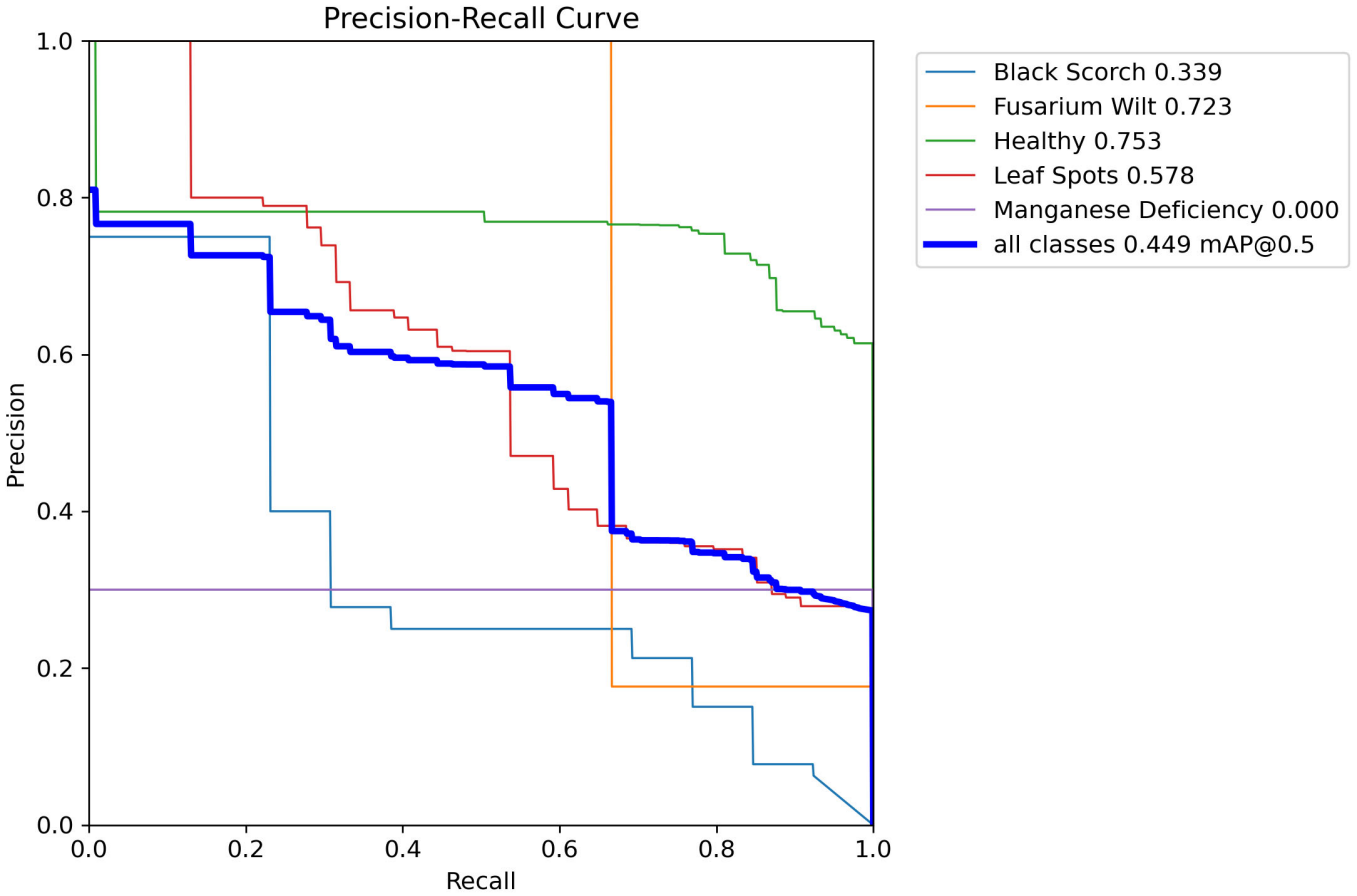
Presents the precision recall curve of date palm leaves dataset.

**Figure 20:**
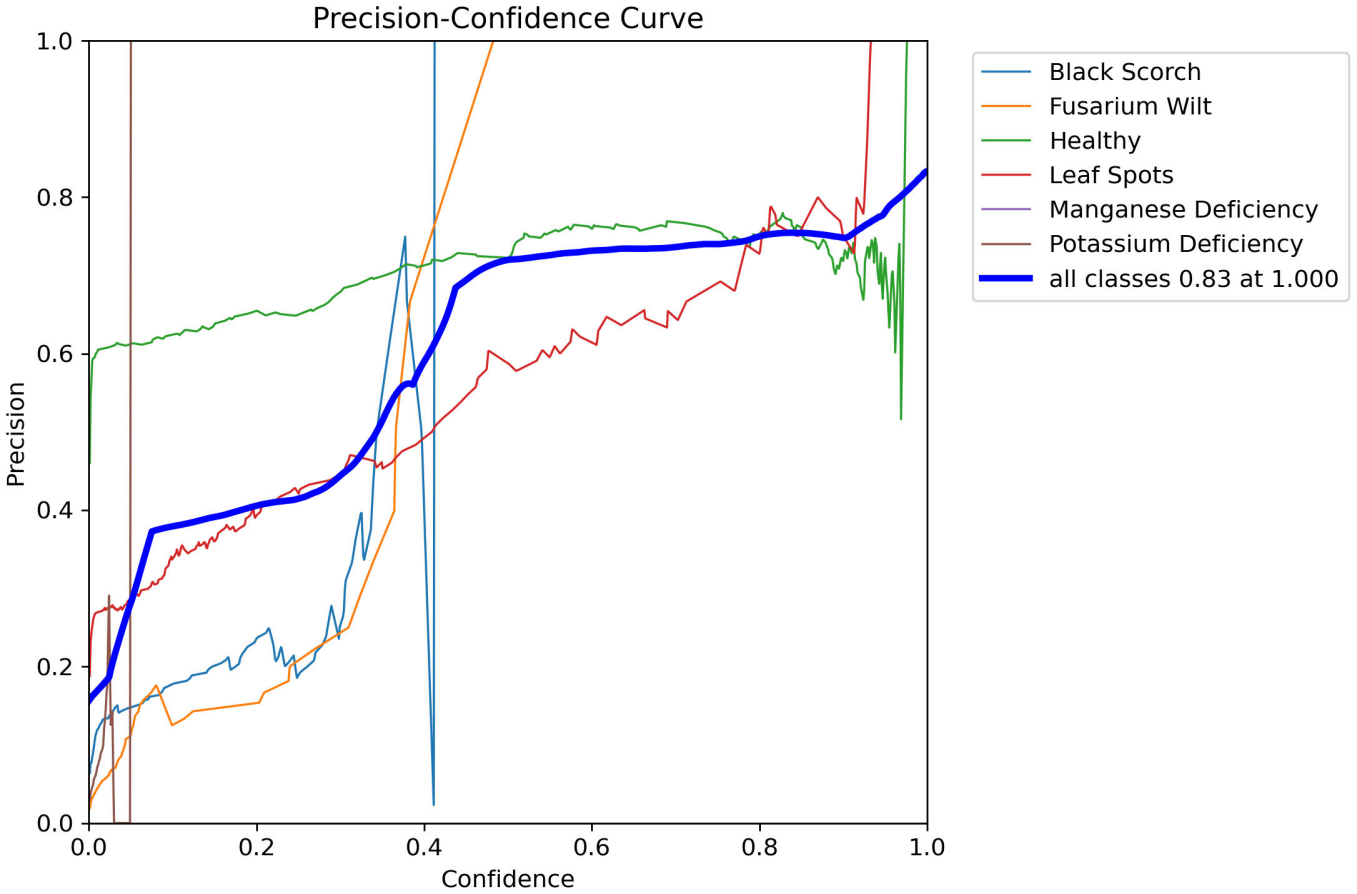
Presents the precision confidence curve of date palm leaves dataset.

The various figures together constitute a full end-to-end workflow for date palm disease analysis, starting from data preparation and going all the way to high-fidelity visual and quantitative outputs. The workflow component we started with is the carefully managed dataset, which we designated for training, validation, and testing. Preprocessing and augmentation were performed on the images to ensure that the models are robust. Each component is integrated into a deep learning pipeline that encompasses high-level classification (Swin Transformer), real-time object detection (YOLOv12 model), and pixel-level segmentation (Grounding DINO + SAM2 module). Given the extent of work conducted to train each of these models properly, we feel that they can satisfactorily perform these tasks, and we give some examples of modeling performance (**Figures 17**–**20**), which show decreasing loss curves and increasing metrics like mAP and F1-score after 156 epochs, confirming effective learning and robust model performance (used for fine verification). It is well known that this systematic workflow will not replace human interaction, and practical aspects will be largely based upon the physical conditions present on the date palms. One key use of this workflow is clearly shown in **Figure 9**, which shows our model detecting and confidently localizing a variety of diseases, regardless of being zero-shot, with diseases including “magnesium deficiency” and “*P. blanchardi*”, outlined toward the bottom of the results. Finally, **Figure 12** illustrates the pipeline’s final capacity for fine-grained analysis, where Grounding DINO’s initial detections are transformed by SAM to precise segmentation masks for all conditions from “potassium deficiency” to “black scorch”, affirming that the full model can provide fine-grained and actionable data.

The integrated performance outcomes in **Table 6** confirm the effectiveness of the proposed hybrid framework. The performance of the classification module was excellent with a 98.91% accuracy, good precision (98.85%), good recall (96.80%), and a good F1-score (96.40%). The performance of the detection module reported a high mAP@0.5 of 97.82%, which confirms reliable object localization. The segmentation results showed an IoU of 92.14% and a Dice score of 93.05%, both of which depict precise pixel-level segmentation of diseased regions. Overall, these findings have demonstrated potentially strong end-to-end effectiveness of this framework through each of the classification, detection, and segmentation tasks, providing a driving benchmark for palm disease research.

**Table 6:** Comparative performance metrics on infected date palm leaves.

Task	Metric(s)	Result (%)
Classification	Accuracy	98.91
	Precision	98.85
	Recall	96.80
	F1-score	96.40
Detection	mAP@0.5	97.82
	mAP@0.5–0.95	–
Segmentation	IoU	92.14
	Dice score	93.05

## 7 Biological relevance and practical implications

### 7.1 Discussion and limitations

The proposed hybrid framework is highly biologically relevant for the basic management of palm diseases. The system allows for the early and accurate detection of infections, which enables farmers and agronomists to conduct timely actions such as the correct application of pesticides, targeted pruning of diseased fronds, or decisions about soil nutrients to limit the spread of the disease. Furthermore, the integrated module for severity quantification can provide a solid decision support system, allowing users to prioritize management actions according to the level of infection, ensuring that resources are used efficiently. Overall, these capabilities are sufficient to measurably reduce losses in revenue, improve accuracy in disease prevention and control, and establish a viable palm evolution system by minimizing unnecessary chemical inputs while centrally protecting yield.

However, there are limitations to consider. To begin with, while zero-shot segmentation with both Grounding DINO and SAM2.1 reduces the extent to which labeled data are required, the actual performance of these models will vary depending on the extent to which the textual cues are clear and specific. For example, with visual disease symptoms that overlap (e.g., magnesium deficiency vs. leaf spots), the model presented prediction ambiguity, as suggested by similar probabilities across multiple classes. This also suggests that better separation between classes and better confidence calibration are also needed. Furthermore, while data augmentation and preprocessing are occurring, there may still be potential sources of class imbalance or workflow omissions that could lead to misclassification or Koch bias with the model, such as environmental noise (dust in images and different lighting regimes) and other sources of intra-class variation. Furthermore, all predictions with the tool are based solely on the visual spatial description of leaf images and do not include any physical data (e.g., temperature, humidity, or nutritional status), which could help the tool achieve greater predictive power, especially concerning early disease detection.

## 8 Conclusion, limitations, and future direction

This study presents a solid and scalable deep learning pipeline designed for automated detection, classification, segmentation, and severity prediction of date palm leaf diseases in a cultivar-independent manner. It achieves this by combining the latest transformer-based architectures, including the Swin Transformer for classification, YOLOv12 for fast object detection, Grounding DINO with SAM2.1 for zero-shot segmentation, and ViTs with a regression head for severity scoring, into a complete and highly accurate solution. Overall, the work provides evidence that this hybrid system can work exceptionally well in laboratory settings and harsh field settings. The classification accuracy of the model captured nearly all the data (98.91%), and the precision and F1-scores surpassed those of many baseline CNN alternatives. The salient contribution of this pipeline is its ability to operate with little or no human labeling, having a fairly straightforward and thus easily replicable (or compatible) set of human and computational costs, and allowing most agricultural practices and environments to allow model validation through either zero-shot or self-supervised learning approaches.

Future directions offer multiple possibilities to enhance and consolidate this research. Implementing XAI tools such as Grad-CAM, LIME, or SHAP would allow farmers and agronomists a way to visualize or interpret the model’s decisions more explicitly, resulting in improved trust and confidence with on-the-ground execution. Including rarer and newly developing diseases, multiple palm species, and inter-regional data can improve model generalizability and reduce overfitting in the conditions the model is trained on. Adding multimodal data and multiple data sources, such as remote sensing imagery, environmental sensors, and weather data, can enable models to make more informed decisions that are contextualized for long-term crop management decisions. Creating a mobile application or drone-based platform that can diagnose diseases in real-time would be another considerable improvement in usability for farmers in remote or resource-limited areas. Lastly, considering longitudinal studies and field trials are the best ways to understand the real-world outcomes of model performance over longer time frames, allowing for developments in fully autonomous, precision agriculture systems to ensure the sustainable management of date palms.

## Data Availability

The raw data supporting the conclusions of this article will be made available by the authors, without undue reservation.
